# Measuring income inequality via percentile relativities

**DOI:** 10.1007/s11135-024-01881-2

**Published:** 2024-05-07

**Authors:** Vytaras Brazauskas, Francesca Greselin, Ričardas Zitikis

**Affiliations:** 1 University of Wisconsin-Milwaukee, Milwaukee, WI USA; 2 Università degli Studi di Milano-Bicocca, Milano, Italy; 3 Western University, London, ON Canada

**Keywords:** Measures of inequality, Heavy-tailed distributions, Income transfers

## Abstract

The adage “the rich are getting richer” refers to increasingly skewed and heavily-tailed income distributions. For such distributions, the mean is not the best measure of the center, but the classical indices of income inequality, including the celebrated Gini index, are mean based. In view of this, it has been proposed in the literature to incorporate the median into the definition of the Gini index. In the present paper we make a further step in this direction and, to acknowledge the possibility of differing viewpoints, investigate three median-based indices of inequality. These indices overcome past limitations, such as: (1) they do not rely on the mean as the center of, or a reference point for, income distributions, which are skewed, and are getting even more heavily skewed; (2) they are suitable for populations of any degree of tail heaviness, and income distributions are becoming increasingly such; and (3) they are unchanged by, and even discourage, transfers among the rich persons, but they encourage transfers from the rich to the poor, as well as among the poor to alleviate their hardship. We study these indices analytically and numerically using various income distribution models. Real-world applications are showcased using capital incomes from 2001 and 2018 surveys from fifteen European countries.

## Introduction

Measuring income inequality has been a challenging task, as each of the indices used for the purpose attempt to condense the complexities of populations into just one number. Among the many indices, we have the Atkinson, Bonferroni, Gini, Palma, Pietra, Theil, and Zenga indices, to name just a few associated with the names of their inventors. Treatises have been written on the topic, such as the handbook by Atkinson and Bourguignon (2000, 2015), which also contains many references to earlier studies. Illuminating monographs on the topic have been written by, for example, Gini (1912), Bonferroni (1930), Kakwani (1980), Nygård and Sandström (1981), Sen (1997, 1998), Champernowne and Cowell (1998), Amiel and Cowell (1999), Atkinson and Piketty (2007), Cowell (2011), Yitzhaki and Schechtman (2013), and Piketty (2014).

The indices are often the areas under certain income-equality curves, which are considerably more difficult to present and explain to the general audience, let alone to easily compare. For example, the Gini index of inequality is 1 minus twice the area under the Lorenz curve. (We shall give mathematical definitions later in this paper). The curves and thus the indices are based on comparing the mean income of the poor with other means, such as the mean income of the entire population, the mean income of the nonpoor, and the mean income of the rich, whatever the definitions of “poor” and “rich” might be. Hence, to be well defined, the curves and the indices inevitably assume that the mean of the underlying population is finite. With the rising income inequality, and thus with the distribution of incomes becoming more skewed and heavily tailed, researchers have therefore sought other ways for measuring inequality.

Gastwirth (2014) proposed to use the median instead of the mean when “normalizing” the absolute Gini mean difference, widely known as the GMD. The author noted, however, that the proposed index might fall outside the class of normalized indices because it compares the *mean* income of the poor with the *median* income of the entire population. There is a natural remedy to this normalization issue: compare the *median* income of the poor with the *median* of the population. Even more, we can compare the median income of the poor with the median of the “not poor” or, for example, with the median of the rich, whatever the latter might mean. This is the path – advocated also by Prendergast and Staudte (2016, 2018), Jokiel-Rokita and Pia̧tek (2023), and Pia̧tek (2023) – that we take in this paper.

In this regard we wish to mention the study of Bennett and Zitikis (2015) where it is shown that a number of classical indices of income inequality arise naturally from a Harsanyi-inspired model of choice under risk, with persons acting as *reference-dependent* expected-utility maximizers in the face of an income quantile lottery, thus giving rise to a reinterpretation of the classical indices as measures of the desirability of redistribution in society. This relativistic approach to constructing indices of income inequality was further explored by Greselin and Zitikis (2018), although more from the modeller’s perspective than from the philosophical one. The present paper, whose preliminary version appeared in the form of a technical report by Brazauskas et al. (2023), further advances this line of research by showing how naturally percentile-based indices arise in this relativistic context, and how they facilitate inequality measurement, especially in the recent-past and current socioeconomic situations. These indices overcome a number of past limitations, such as:

- They do not rely on the mean as the center of, or a reference point for, income distributions, which are skewed, and are getting even more heavily skewed.
- They are suitable for populations of any degree of tail heaviness, and income distributions are becoming increasingly such.
- They are unchanged by, and even discourage, transfers among the rich persons, but they encourage transfers from the rich to the poor, as well as among the poor to alleviate their hardship.

In more technical terms, while analyzing capital incomes, Greselin et al. (2014) observed that the Hill estimator of the tail index yields a value in the interval (0.5, 1) for some of the samples. In such cases, the classical mean-based inequality indices are inappropriate, as the mean does not exist. Consequently, these empirical observations prompted us to seek new approaches, as we do in the present paper, for measuring economic/income inequality that are suitable for all distributions, irrespective of their tail heaviness.

The rest of the paper is organized as follows. In Sect. 2 we define the inequality indices, alongside the corresponding equality curves, preceded by several classical indices for comparison purposes. In Sect. 3 we illustrate the indices and their curves numerically, using several popular families of distributions. In Sect. 4, we use the indices to first analyze capital incomes of European countries using data from a 2001 survey, and then we compare the results with those obtained from a 2018 survey. In Sect. 5 we look at the indices from the perspective of income transfers. Sect. 6 concludes the paper with a brief recap. Proofs and other technicalities are in Appendix 1.

## Inequality indices and their curves

We start with technical prerequisites. Let *F* be the cumulative distribution function of the population incomes *X*, a random variable. We assume that *F* is non-negatively supported, that is, $F(x)=0$ for all real $x<0$. Furthermore, let *Q* denote the (generalized) inverse of *F*, called the quantile function. That is, for each $p\in (0,1)$, *Q*(*p*) is the smallest number *x* such that $F(x)\ge p$. Hence, the population median income is

$$\begin{aligned} m=Q(1/2), \end{aligned}$$

assumed throughout this paper to be strictly greater than 0. Generally, *Q*(*p*) is the $p\times 100^{\textrm{th}}$ percentile. Furthermore, the median income of the poorest $p\times 100\%$ persons is *Q*(*p*/2). Based on these quantities, we shall later describe three ways for measuring inequality, but first, we recall the definitions of a few classical indices that serve as benchmarks for our current study.

### In the classical mean-based world

The index of Gini (1914) is the most widely-used measure of inequality. It can be expressed in a myriad of ways (e.g., Yitzhaki 1998; Yitzhaki and Schechtman 2013). For example, the Gini index can be written in terms of the Bonferroni curve

$$\begin{aligned} b(p)={1\over \mu p }\int _0^p Q(s)\,\textrm{d}s, \quad 0 \le p \le 1, \end{aligned}$$

as follows:

2.1 $$\begin{aligned} G&= 2\int _0^1 \bigg (1-{{1\over p}\int _0^p Q(s)\,\textrm{d}s \over \mu } \bigg )p \,\textrm{d}p \nonumber \\&= 1- \int _0^1 {{1\over p}\int _0^p Q(s)\,\textrm{d}s \over \mu } 2p \,\textrm{d}p \nonumber \\&= 1-\int _0^1 b(p)\, 2p \,\textrm{d}p, \end{aligned}$$

where

$$\begin{aligned} \mu =\int _0^1 Q(s)\,\textrm{d}s \end{aligned}$$

is the mean of *X*, assumed in this section to be finite and strictly greater than 0.

Zenga (2007) argued that the mean income of those below the percentile *Q*(*p*) need to be compared not with the mean of all the incomes but with the mean income of those above the percentile *Q*(*p*). This point of view led the author to the index

$$\begin{aligned} Z =1-\int _0^1 {{1\over p}\int _0^p Q(s)\,\textrm{d}s \over {1\over 1-p}\int _p^1 Q(s)\,\textrm{d}s } \,\textrm{d}p. \end{aligned}$$

Davydov and Greselin (2019, 2020) suggested to modify Zenga’s idea by comparing the mean income of those below the percentile *Q*(*p*) with the mean income of those above the percentile $Q(1-p)$. This point of view led the authors to the index

$$\begin{aligned} D =1-\int _0^1 {{1\over p}\int _0^p Q(s)\,\textrm{d}s \over {1\over p}\int _{1-p}^1 Q(s)\,\textrm{d}s } \,\textrm{d}p. \end{aligned}$$

Of course, 1/*p* in the numerator and denominator cancel out, but in this way written *D* facilitates an easier comparison with *Z*.

### A transition into the heavily tailed modern world

Unlike the above three mean-based indices *G*, *Z* and *D*, the index of Gastwirth (2014) is a mean-median based index. Namely, given the well-known expression

2.2 $$\begin{aligned} G= {\text {GMD} \over 2 \mu } \end{aligned}$$

of the Gini index *G* in terms of the Gini mean difference (GMD), which is often written as the expectation $\mathbb {E}(|X_1-X_2|)$, where $X_1$ and $X_2$ are two independent copies of *X*, Gastwirth (2014) argued that comparing the GMD with twice the median would be better than comparing with twice the mean as in Eq. (2.2). This viewpoint has given rise to the index

$$\begin{aligned} G_2&= {\text {GMD} \over 2 m }\\&= \int _0^1 \bigg ( {\mu \over m} -{{1\over p}\int _0^p Q(s)\,{\text d}s \over m}\bigg )2p\,\textrm{d}p\\&= {\mu \over m}-\int _0^1 {{1\over p}\int _0^p Q(s)\,{\text d}s \over m} 2p\,\textrm{d}p . \end{aligned}$$

Note that $\mu /m$, which can be viewed as the benchmark replacing 1 in the previous indices, is the mean-median ratio that has been used as an easy to understand – and thus to convey to the general audience – indicator of wealth and income distribution (e.g., Garratt 2020). In the case of symmetric distributions, $\mu /m$ is of course equal to 1.

### In the skewed and heavily tailed modern world: quantile-based indices

The above discussion naturally leads to three strategies of defining purely median-based indices of income inequality and their corresponding curves of equality, all based on percentiles and thus well defined irrespective of whether the income variable *X* has a finite first or any other moment.

#### Strategy 1

**Fig. 1:**
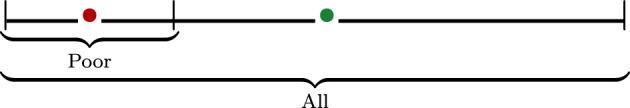
The median of the poor (red) and the median of all (green). (Color figure online)

Compare the median income of the poorest $p\times 100\%$ persons with the median of the entire population (Fig. 1).

This leads to the equality curve

2.3 $$\begin{aligned} \psi _1(p) = {Q(p/2) \over Q(1/2)}, \quad 0<p<1, \end{aligned}$$

also independently introduced by Jokiel-Rokita and Pia̧tek (2023, Eq. (9)). Compare it also with $L_1(F;p)$ of Prendergast and Staudte (2016, Definition 1). Averaging this curve over all *p*’s gives rise to the inequality index

2.4 $$\begin{aligned} \Psi _1= 1-\int _{0}^{1} {Q(p/2) \over Q(1/2)} \,\textrm{d}p. \end{aligned}$$

Compare it with the left-most integral of Prendergast and Staudte (2016, Eq. (3)). Note the mathematical similarity between the Bonferroni curve *b* and the curve $\psi _1$:

$$\begin{aligned} b(p)={{1\over p}\int _0^p Q(s)\,\textrm{d}s \over \int _0^1 Q(s)\,\textrm{d}s}, \quad \psi _1(p) ={{1\over p}\int _0^p Q(p/2)\,\textrm{d}s \over \int _0^1 Q(1/2)\,\textrm{d}s}. \end{aligned}$$

#### Strategy 2

**Fig. 2:**
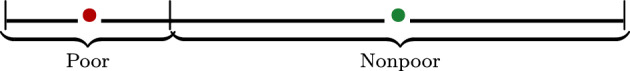
The median of the poor (red) and the median of the nonpoor (green). (Color figure online)

Compare the median income of the poorest $p\times 100\%$ persons with the median of the nonpoor (Fig. 2).

This leads to the equality curve

2.5 $$\begin{aligned} \psi _2(p) = {Q(p/2) \over Q(1/2+p/2)}, \quad 0<p<1, \end{aligned}$$

also independently introduced by Jokiel-Rokita and Pia̧tek (2023, Eq. (10)), which is well defined because we assume that the median income $m=Q(1/2)$ is strictly positive, and thus $Q(1/2+p/2)$, being not smaller than *Q*(1/2), is strictly positive for every $0<p<1$. Averaging this curve over all *p*’s gives rise to the inequality index

2.6 $$\begin{aligned} \Psi _2= 1-\int _{0}^{1} {Q(p/2) \over Q(1/2+p/2)}\, \textrm{d}p, \end{aligned}$$

which is also considered by Pia̧tek (2023). Note the mathematical similarity between the Zenga curve *z* and the curve $\psi _2$:

$$\begin{aligned} z(p)= {{1\over p}\int _0^p Q(s)\,\textrm{d}s \over {1\over 1-p}\int _p^1 Q(s)\,\textrm{d}s }, \quad \psi _2(p) = {{1\over p}\int _0^p Q(p/2)\,\textrm{d}s \over {1\over 1-p}\int _p^1 Q(p+(1-p)/2)\,\textrm{d}s}. \end{aligned}$$

#### Strategy 3

**Fig. 3:**
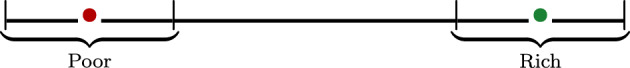
The median of the poor (red) and the median of the rich (green). (Color figure online)

Compare the median income of the poorest $p\times 100\%$ persons with the median of the richest $p\times 100\%$ persons (Fig. 3).

This leads to the equality curve (Prendergast and Staudte 2018)

2.7 $$\begin{aligned} \psi _3(p) = {Q(p/2) \over Q(1-p/2)}, \quad 0<p<1, \end{aligned}$$

also considered by Jokiel-Rokita and Pia̧tek (2023, Eq. (11)), which is well defined because we assume that the median income $m=Q(1/2)$ is strictly positive, and thus $Q(1-p/2)$, being not smaller than *Q*(1/2), is strictly positive for every $0<p<1$. Compare this curve also with $L_2(F;p)$ of Prendergast and Staudte (2016, Definition 1). Averaging this curve over all *p*’s gives rise to the inequality index (Prendergast and Staudte 2018)

2.8 $$\begin{aligned} \Psi _3= 1-\int _{0}^{1} {Q(p/2) \over Q(1-p/2)} \,\textrm{d}p, \end{aligned}$$

which is also considered by Pia̧tek (2023). Note the mathematical similarity between the Davydov-Greselin curve *d* and the curve $\psi _3$:

$$\begin{aligned} d(p)= {{1\over p}\int _0^p Q(s)\,\textrm{d}s \over {1\over p}\int _{1-p}^1 Q(s)\,\textrm{d}s }, \quad \psi _3(p) = {{1\over p}\int _0^p Q(p/2)\,\textrm{d}s \over {1\over p}\int _{1-p}^1 Q(1-p+p/2)\,\textrm{d}s}. \end{aligned}$$

#### A recap

Summarizing the above discussion, in view of Eqs. (2.4), (2.6), and (2.8), the three income-equality curves are connected to the corresponding income-inequality indices via the equation

2.9 $$\begin{aligned} \Psi _k= 1-\int _{0}^{1} \psi _k(p)\,\textrm{d}p. \end{aligned}$$

Note that the three curves $\psi _k$ take values only in the interval [0, 1], and so the three indices $\Psi _k$ are always normalized, that is, $\Psi _k \in [0,1]$. In this context it is useful to look at the following unrealistic cases:

- If the income-equality curve $\psi _k$ is equal to 1 everywhere on (0, 1), which implies quantile-based income equality, then the income-inequality index $\Psi _k$ is equal to 0, which means lowest inequality.
- If the income-equality curve $\psi _k$ is equal to 0 everywhere on (0, 1), which implies extreme quantile-based income inequality, then the income-inequality index $\Psi _k$ is equal to 1, which means maximal inequality.

For a cautionary and illuminating note concerning the meaning of quantile-based income equality and extreme quantile-based income inequality, we shall have Example 1 below.

Hence, these two extreme cases serve as benchmark curves: the one that is identically equal to 1 is the curve of perfect equality, and the one that is identically equal to 0 is the curve of extreme inequality. We can therefore say that the three indices $\Psi _k$ measure the deviation of the actual curves $\psi _k$ from the benchmark egalitarian curve $\psi _e(p)=1$, $0\le p \le 1$, by calculating the areas between them.

##### Example 1

Consider a society with $$n\ge 3$$ subjects, one of which is the ruler. Each of the working $$n-1$$ subjects earns $ 1, just to be taken away by the ruler. Hence, ultimately, each of the working subjects possesses $ 0 and the ruler has $ $$(n-1)$$. This is a textbook example of extreme inequality.

The classical income inequality indices are mean based, which is not – as argued by statisticians – an appropriate measure of the center in the case of skewed populations. When, on the other hand, the median is used to measure the center, the large values, such as the income of the ruler in the above society, do not influence the center. Indeed, the center of incomes in the above society is $ 0, which is a more appropriate description of the typical income than the mean value $$\$\,(n-1)/n$$ would be. (The ruler does not work and accumulates wealth only by taking away $ 1 from each of the working subjects).

Hence, in summary, we can say that the mean-based society views the ruler as a member of the society, making $\$\,(n-1)/n$ a typical value of the society, whereas the median-based society views the ruler as being above the society, that is, not in the society, and so the typical income in this case is $\$\,0$.

To see what happens with the three quantile-based indices $\Psi _k$ and their curves $\psi _k$ in the above situation of “extreme inequality,” we first recall that we have assumed that the median must be above zero. To accommodate this condition, we assume that the ruler lets each of the $n-1$ working subjects keep a small amount $\$\,\varepsilon \in (0,1)$ of their earned $\$\,1$. Hence, the ruler accumulates the wealth of $\$\,(n-1)(1-\varepsilon )$, making sure – needless to say – that the ruler’s wealth is not smaller than that of any of the working subjects, that is, the inequality $(n-1)(1-\varepsilon )\ge \varepsilon$ holds. Note that this inequality is equivalent to $\varepsilon \le 1-1/n$, thus implying – quite naturally – that the more subjects there are in the society, the larger the amount they can be allowed to retain for their own use without making them richer than the ruler.

The quantile function *Q* in this scenario is

$$\begin{aligned} Q(u)= \left\{ \begin{array}{ll} \varepsilon &{} \hbox { for} \quad 0<u\le {n-1\over n}, \\ (n-1)(1-\varepsilon ) &{} \hbox { for} \quad {n-1\over n}<u\le 1, \end{array} \right. \end{aligned}$$

and thus the median income is $Q(1/2)=\varepsilon$. We have the following expressions:

$$\begin{aligned} \psi _1(p)&={\varepsilon \over \varepsilon }=1 &\Longrightarrow \quad \Psi _1=0,\\ \psi _2(p)&= \left\{ \begin{array}{ll} 1 &{} \hbox { for} \quad p\le 1-{2\over n} \\ {\varepsilon \over (n-1)(1-\varepsilon )} &{} \hbox { for} \quad p>1-{2\over n} \end{array} \right. &\Longrightarrow \quad \Psi _2={2\over n} \bigg (1-{\varepsilon \over (n-1)(1-\varepsilon )} \bigg ),\\ \psi _3(p)&= \left\{ \begin{array}{ll} {\varepsilon \over (n-1)(1-\varepsilon )} &{} \hbox { for} \quad p<{2\over n} \\ 1 &{} \hbox { for} \quad p\ge {2\over n} \end{array} \right. &\Longrightarrow \quad \Psi _3={2\over n} \bigg ( 1-{\varepsilon \over (n-1)(1-\varepsilon )} \bigg ). \end{aligned}$$

Note that the inequality $(n-1)(1-\varepsilon )\ge \varepsilon$ ensures that the indices $\Psi _2$ and $\Psi _3$ are non-negative, just like the index $\Psi _1=0$ is. In the case $(n-1)(1-\varepsilon )= \varepsilon$, we have quantile-based income equality, that is, $\psi _2(p)=1$ and $\psi _3(p)=1$ for all $p\in (0,1)$, and thus $\Psi _2=0$ and $\Psi _3=0$.

Hence, the index $\Psi _1$ indicates equality in the aforementioned society (recall that the ruler is above the society, not in it), whereas the indices $\Psi _2$ and $\Psi _3$, each indicating some degree of inequality when $(n-1)(1-\varepsilon )> \varepsilon$, show that the inequality ultimately vanishes when the society grows in size, that is, when $n\rightarrow \infty$. This concludes Example 1.

## The indices and curves: a parametric viewpoint

Modelling population incomes using parametric distributions and also fitting such distributions to income data are common approaches in the area (e.g., Kleiber and Kotz 2003). From this perspective, the inequality indices *G*, *Z*, *D* and $G_2$ and their corresponding equality curves have been amply discussed and illustrated by their inventors and subsequent researchers. Hence, we devote this section to illustrating only the three indices $\Psi _k$ and their corresponding curves $\psi _k$.

We use nine parametric families of distributions, most of which are common in modeling incomes (e.g., Kleiber and Kotz 2003). They are right skewed and present a full spectrum of tail heaviness: some are lightly tailed (e.g., exponential), some are heavily tailed (e.g., Pareto distributions), and others have the right tails of intermediate heaviness (e.g., lognormal). For their specific parametrizations, Table 1 contains all the essential formulas.

**Table 1:** The quantile function *Q* and the income equality functions $\psi _k$ for selected parametric distributions

Distributions	*Q*(*u*)	$\psi _1(p)$	$\psi _2(p)$	$\psi _3(p)$
Uniform$(0, \theta )$	$\theta u$	*p*	$\frac{p}{1+p}$	$\frac{p}{2-p}$
Exponential$(0, \theta )$	$-\theta \log (1-u)$	$\frac{\log (1-p/2)}{\log (1/2)}$	$\frac{\log (1-p/2)}{\log (1+p/2)}$	$\frac{\log (1-p/2)}{\log (p/2)}$
Gamma$(\theta , \alpha )$	$\theta \, \Gamma ^{-1}_{\alpha }(u)$	$\frac{\Gamma ^{-1}_{\alpha }(p/2)}{\Gamma ^{-1}_{\alpha }(1/2)}$	$\frac{\Gamma ^{-1}_{\alpha }(p/2)}{\Gamma ^{-1}_{\alpha }(1/2+p/2)}$	$\frac{\Gamma ^{-1}_{\alpha }(p/2)}{\Gamma ^{-1}_{\alpha }(1-p/2)}$
Weibull$(\theta , \tau )$	$-\theta \big ( \log (1-u) \big )^{1/\tau }$	$\left( \frac{\log (1-p/2)}{\log (1/2)} \right) ^{1/\tau }$	$\left( \frac{\log (1-p/2)}{\log (1+p/2)} \right) ^{1/\tau }$	$\left( \frac{\log (1-p/2)}{\log (p/2)} \right) ^{1/\tau }$
Lognormal$(\mu , \sigma )$	$e^{\mu + \sigma \Phi ^{-1}(u)}$	$e^{\sigma \, \Phi ^{-1}(p/2)}$	$\left( \frac{e^{\Phi ^{-1}(p/2)}}{e^{\Phi ^{-1}((1+p)/2)}} \right) ^{\sigma }$	$e^{2 \sigma \, \Phi ^{-1}(p/2)}$
Log-Cauchy$(\mu , \sigma )$	$e^{\mu + \sigma \tan (\pi (u-1/2))}$	$e^{\sigma \tan ( \pi (p-1)/2)}$	$\left( \frac{e^{\tan (\pi (p-1)/2)}}{e^{\tan (\pi p/2)}} \right) ^{\sigma }$	$e^{2 \sigma \tan ( \pi (p-1)/2)}$
Pareto-II$(\sigma , \alpha )$	$\sigma \big ( (1-u)^{-1/\alpha } - 1 \big )$	$\frac{(1-p/2)^{-1/\alpha }-1}{(1/2)^{-1/\alpha }-1}$	$\frac{(1-p/2)^{-1/\alpha }-1}{((1-p)/2)^{-1/\alpha }-1}$	$\frac{(1-p/2)^{-1/\alpha }-1}{(p/2)^{-1/\alpha }-1}$
Pareto-III$(\sigma , \gamma )$	$\sigma \big ( (1-u)^{-1} - 1 \big )^{\gamma }$	$\left( \frac{p}{2-p} \right) ^{\gamma }$	$\left( \frac{p(1-p)}{(1+p)(2-p)} \right) ^{\gamma }$	$\left( \frac{p}{2-p} \right) ^{2 \gamma }$
Pareto-IV$(\sigma , \alpha , \gamma )$	$\sigma \big ( (1-u)^{-1/\alpha } - 1 \big )^{\gamma }$	$\left( \frac{(1-p/2)^{-1/\alpha }-1}{(1/2)^{-1/\alpha }-1} \right) ^{\gamma }$	$\left( \frac{(1-p/2)^{-1/\alpha }-1}{((1-p)/2)^{-1/\alpha }-1} \right) ^{\gamma }$	$\left( \frac{(1-p/2)^{-1/\alpha }-1}{(p/2)^{-1/\alpha }-1} \right) ^{\gamma }$

Note 1: $\Gamma ^{-1}_{\alpha }(u)$ denotes the quantile function of the *Gamma*$(\theta = 1, \alpha )$ distribution

Note 2:  $\Phi ^{-1}(u)$ denotes the quantile function of the *Normal*$(\mu = 0, \sigma = 1)$ distribution

We have computed the inequality indices $\Psi _k$ for these distributions under various parameter choices, to be clarified and discussed in a moment. The results are in Table 2,

**Table 2:** The inequality indices $\Psi _k$ for various parametric distributions and the rankings of these distributions based on the indices

Distributions	Inequality indices			Ranks based on		
	$\Psi _1$	$\Psi _2$	$\Psi _3$	$\Psi _1$	$~~\Psi _2$	$\Psi _3$
Uniform$(0, \theta )$	0.5000	0.6936	0.6147	6	2	3–4
Exponential$(0, \theta )$	0.5583	0.8327	0.7026	7	7	7
Gamma$(\theta , \alpha = 0.5)$	0.6874	0.9378	0.8020	12	10	11
Gamma$(\theta , \alpha = 2)$	0.4360	0.6974	0.5956	3	3	2
Weibull$(\theta , \tau = 0.5)$	0.7237	0.9681	0.8358	13	13	13
Weibull$(\theta , \tau = 2)$	0.3810	0.6022	0.5239	1	1	1
Lognormal$(\mu , \sigma = 1)$	0.4779	0.7886	0.6648	4	5	5
Lognormal$(\mu , \sigma = 2)$	0.6648	0.9527	0.8122	11	12	12
Log-Cauchy$(\mu , \sigma = 1)$	0.6054	0.9382	0.7470	9	11	9
Log-Cauchy$(\mu , \sigma = 2)$	0.7470	0.9935	0.8551	14	16	14
Pareto-II$(\sigma , \alpha = 1)$	0.6147	0.9242	0.7736	10	9	10
Pareto-II$(\sigma , \alpha = 2)$	0.5868	0.8863	0.7407	8	8	8
Pareto-III$(\sigma , \gamma = 0.5)$	0.4302	0.7344	0.6147	2	4	3–4
Pareto-III$(\sigma , \gamma = 2)$	0.7736	0.9932	0.8795	16	15	16
Pareto-IV$(\sigma , \alpha = 0.5, \gamma = 0.5)$	0.4803	0.8288	0.6887	5	6	6
Pareto-IV$(\sigma , \alpha = 2, \gamma = 2)$	0.7495	0.9852	0.8598	15	14	15

where we also report the rankings of the distributions based on the indices: rank 1 corresponds to the lowest inequality and rank 16 to the highest inequality. It is encouraging to see that while the magnitudes of the indices differ, the rankings induced by them are fairly similar.

**Fig. 4:**
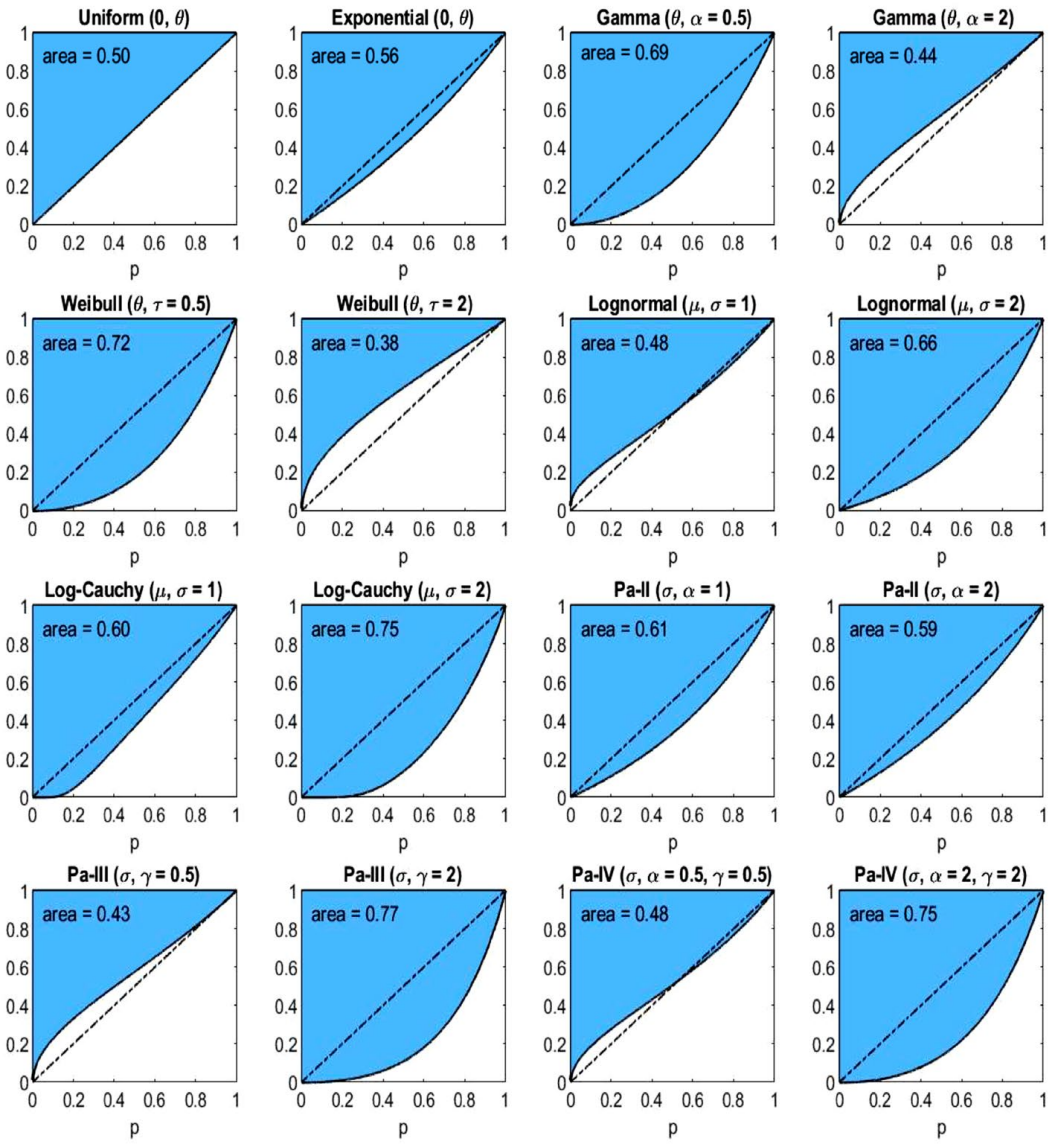
The income-equality curve $\psi _1$ and the shaded-in area (i.e., $\Psi _1$) above it for the distributions of Table 2, with the dash-dotted line depicting $\psi _1$ of the uniform distribution

**Fig. 5:**
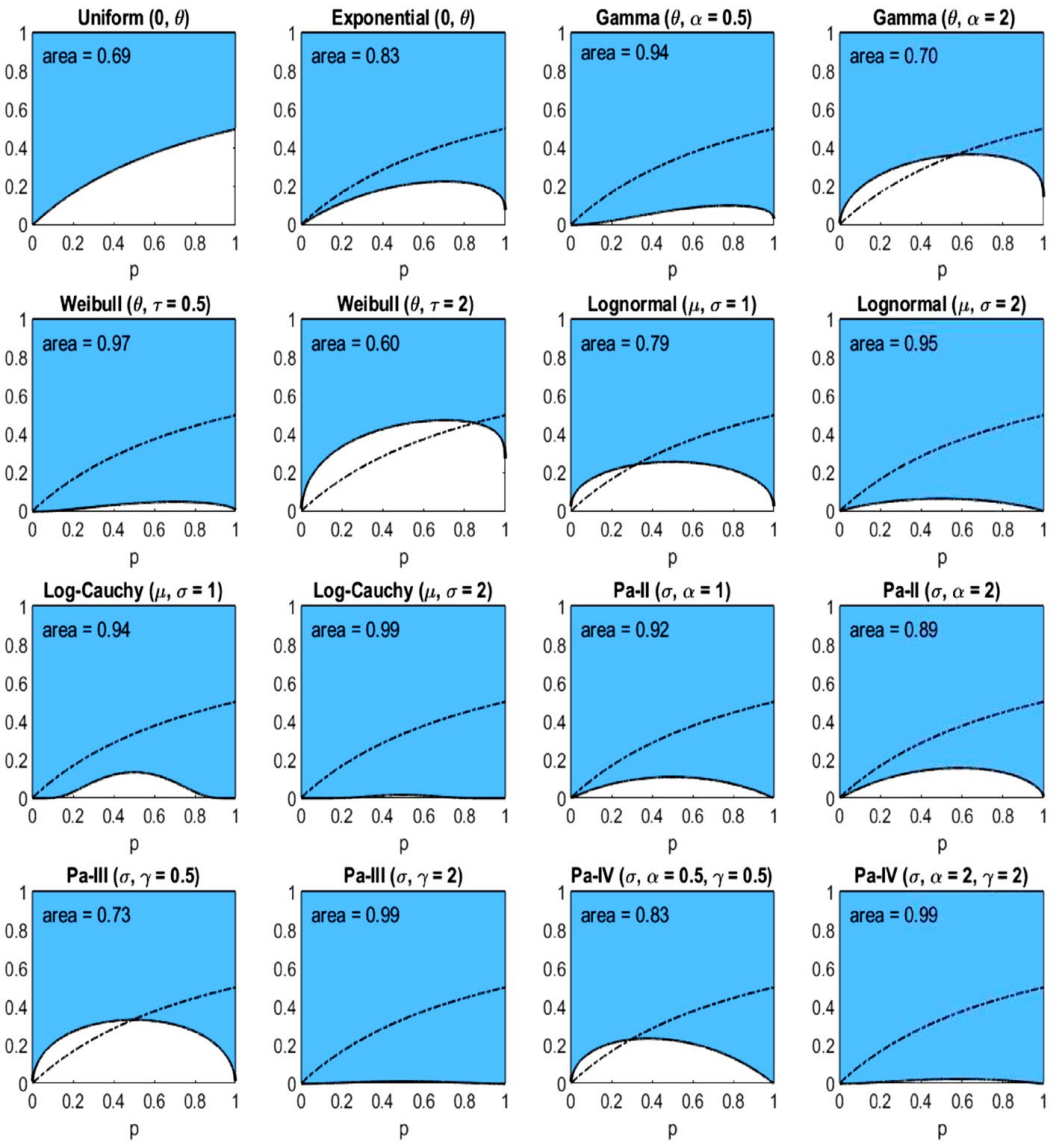
The income-equality curve $\psi _2$ and the shaded-in area (i.e., $\Psi _2$) above it for the distributions of Table 2, with the dash-dotted line depicting $\psi _2$ of the uniform distribution

**Fig. 6:**
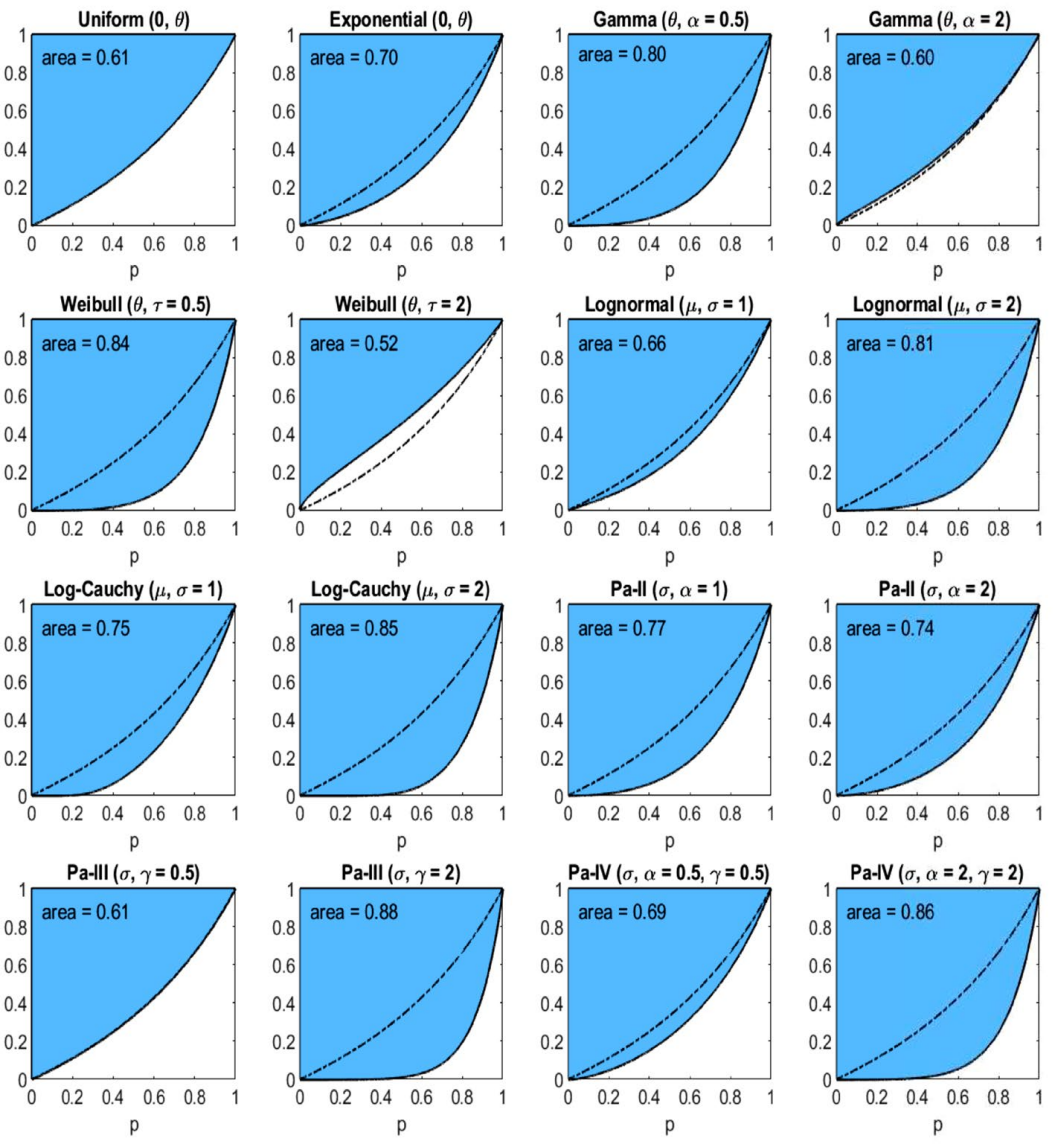
The income-equality curve $\psi _3$ and the shaded-in area (i.e., $\Psi _3$) above it for the distributions of Table 2, with the dash-dotted line depicting $\psi _3$ of the uniform distribution

In Table 2 we have four groups consisting of four distributions. The groups reflect the fact that in Figs. 4, 5, 6, the distributions are grouped into four rows each containing four panels. The figures depict the three income-equality curves $\psi _k$ for the distributions specified in Table 2.

Since the curves are ratios of percentiles, the scale parameter of each distribution has no effect on the inequality indices. The same is true for the log-location parameter ($e^{\mu }$) of the lognormal and log-Cauchy distributions. However, the shape ($\alpha$, $\gamma$) and the log-scale ($e^{\sigma }$) parameters are the primary drivers of the underlying inequality. To explore this effect, we choose a couple of values of each of these parameters for plotting. In particular, since the gamma and Weibull distributions are generalizations of the exponential distribution, it is of interest to see the effect of heavier ($\alpha = \tau = 0.5$) and lighter ($\alpha = \tau = 2$) than exponential tails. Likewise, for the lognormal and log-Cauchy distributions, $\sigma = 1$ represents a “standard” case while $\sigma = 2$ is a heavier-than-standard-tail case. And for the Pareto distributions, the tail heaviness is controlled by the shape parameter $\alpha$: the model has infinite variance when $\alpha \le 2$ and infinite mean when $\alpha \le 1$. Therefore, it makes sense to choose $\alpha$’s around these important benchmarks. In the plots of Figs. 4, 5, 6, the uniform distribution serves as a benchmark for comparing the curves. In each plot, the dash-dotted line marks the curve $\psi _k$ in the case of the uniform distribution. Numerical evaluations labeled ‘area’ represent the areas of the corresponding shaded regions above the curves $\psi _k$, which are the values of the inequality indices.

From Table 2 and Figs. 4, 5, 6 we observe several facts, which follow immediately from the formulas of Table 1:

- $\psi _1$ for *Pareto-III*$(\sigma , \gamma = 2)$ and $\psi _3$ for *Pareto-II*$(\sigma , \alpha = 1)$ coincide, being equal to $(\frac{p}{2-p})^2$, thus giving identical inequality indices 0.7736.
- $\psi _1$ for *Pareto-II*$(\sigma , \alpha = 1)$ and $\psi _3$ for both *Uniform*$(0, \theta )$ and *Pareto-III*$(\sigma , \gamma = 0.5)$ coincide, being equal to $\frac{p}{2-p}$, thus giving identical inequality indices 0.6147.
- $\psi _1$ for *Lognormal*$(\mu , \sigma = 2)$ and $\psi _3$ for *Lognormal*$(\mu , \sigma = 1)$ coincide, being equal to $e^{2 \Phi ^{-1}(p/2)}$, thus giving identical inequality indices 0.6648.
- $\psi _1$ for *Log-Cauchy*$(\mu , \sigma = 2)$ and $\psi _3$ for *Log-Cauchy*$(\mu , \sigma = 1)$ coincide, being equal to $e^{2 \tan (\pi (p-1)/2)}$, thus giving identical inequality indices 0.7470.

We conclude this section with the note that there are, of course, many other parametric distributions for modelling incomes (see, e.g., Kleiber and Kotz 2003).

## A nonparametric viewpoint

We now consider nonparametric (also called empirical) ways for estimating all the aforementioned indices of inequality and their corresponding equality curves, with analyses of real data.

###  Empirical estimators of the quantile-based indices

Let $X_{1},\dots , X_{n}$ denote incomes of randomly selected persons, with $X_{1:n}\le \cdots \le X_{n:n}$ denoting the ordered incomes. We assume that the empirical median

$$\begin{aligned} Q_n(1/2)=X_{\lceil n/2 \rceil :n} \end{aligned}$$

is strictly positive, where, for every real $x\ge 0$, $\lceil x \rceil$ is the smallest integer that is not below *x*. The empirical counterparts of the three indices $\Psi _k$ are (see their justifications in Appendix 1)

4.1 $$\begin{aligned} \Psi _{1,n}&= 1- {1\over \lfloor n/2 \rfloor } \sum _{k=1}^{\lfloor n/2 \rfloor } {X_{k:n} \over X_{\lceil n/2 \rceil :n}} , \end{aligned}$$

4.2 $$\begin{aligned} \Psi _{2,n}&= 1- {1\over \lfloor n/2 \rfloor } \sum _{k=1}^{\lfloor n/2 \rfloor } {X_{k:n} \over X_{\lceil n/2 \rceil +k :n}} , \end{aligned}$$

4.3 $$\begin{aligned} \Psi _{3,n}&= 1- {1\over \lfloor n/2 \rfloor } \sum _{k=1}^{\lfloor n/2 \rfloor } {X_{k:n} \over X_{n-k+1 :n}}, \end{aligned}$$

where, for every real $x\ge 0$, $\lfloor x \rfloor$ is the largest integer that does not exceed *x*. Note that all the three indices are well defined because we assume that the median $X_{\lceil n/2 \rceil :n}$ is strictly positive. When it is desirable to emphasize the dependence of the indices on incomes, we do so by writing them as $\Psi _{k,n}(\textbf{X})$, where $\textbf{X}=(X_{1:n}, \dots , X_{n:n})$ is the vector of all the (ordered) incomes in the sample. Next are a few immediate consequences of definitions (4.1)–(4.3).

#### Property 1

For every real $c\ge 0$, we have $\Psi _{k,n}(c\textbf{X})=\Psi _{k,n}(\textbf{X})$.

This property implies, for example, that changing the currency with which the incomes are reported does not affect the values of the three inequality indices.

#### Property 2

We have the inequality $\Psi _{k,n}(\textbf{X})\ge \Psi _{k,n}(\textbf{X}+c )$ for every real $c\ge 0$. The inequality is strict under the following two conditions: first, $c>0$, and second, there is at least one ratio inside the sum of the definition of $\Psi _{k,n}$ that is not equal to 1. (Note that none of the ratios exceeds 1).

This property implies that adding the same amount of income to everybody does not increase inequality and, under a minor caveat specified in the property, the index even decreases. To see the necessity of the assumption, consider the case when all *X*’s are equal, which gives $\Psi _{k,n}(\textbf{X})=0$ and also $\Psi _{k,n}(\textbf{X}+c )=0$ irrespective of the value of *c*. For a proof of Property 2, as well as for proofs of other properties, see Appendix 1.

#### Property 3

When $c\rightarrow \infty$, we have $\Psi _{k,n}(\textbf{X}+c ) \rightarrow 0$.

Intuitively, this property says that if we keep adding the same positive amount of income to everyone, all else being equal, then we shall eventually eliminate the inequality.

###  Estimators of the mean-based indices

Next we report the definitions of the empirical estimators of *Z*, *D*, *G* and $G_2$ obtained by replacing the population quantile function *Q* by the empirical quantile function $Q_n$, which is given by the equation

4.4 $$\begin{aligned} Q_n(p)=X_{\lceil np \rceil :n} \end{aligned}$$

for every $p\in (0,1]$. Slightly modifying the obtained expression in an asymptotically equivalent way to make it intuitively and computationally more appealing, we arrive at the estimator

$$\begin{aligned} Z_n=1-{1\over n}\sum _{i=1}^{n-1} {{1\over i}\sum _{k=1}^{i}X_{k:n} \over {1\over n-i}\sum _{k=i+1}^{n} X_{k:n}} \end{aligned}$$

of *Z*, which appears in Greselin and Pasquazzi (2009). Likewise, we arrive at

$$\begin{aligned} D_n=1-{1\over n}\sum _{i=1}^{n} {{1\over i}\sum _{k=1}^{i}X_{k:n} \over {1\over i}\sum _{k=n-i+1}^{n} X_{k:n}}, \end{aligned}$$

which is an empirical estimator of *D* that appears in Davydov and Greselin (2020). (Of course, 1/*i* in the numerator and denominator cancel out). The same reasoning leads to the empirical Gini index

$$\begin{aligned} G_n&=1-{2\over n}\sum _{i=1}^{n} {\sum _{k=1}^{i}X_{k:n} \over \sum _{k=1}^{n} X_{k:n}} +{1\over n}\\&= 1-{1\over \overline{X} n^2}\sum _{i=1}^n \big (2(n-i)+1\big ) X_{i:n}, \end{aligned}$$

where the last equation follows from simple algebra, with $\overline{X}$ denoting the mean, assumed to be strictly positive, of the incomes $X_{1},\dots , X_{n}$. Note that the last expression for $G_n$ is the one that places the empirical Gini index into the family of *S*-Gini indices introduced by Donaldson and Weymark (1980) and Weymark (1980/81); see also Zitikis and Gastwirth (2002) for further references and statistical inference.

#### Note 1

The asymptotically negligible term 1/*n* on the right-hand side of the first equation of $G_n$ ensures that $G_n$ makes sense for all sample sizes. Without this term we may get counterintuitive values. For example, when the ‘incomes’ are $X_1=1$, $X_2=2$ and $X_3=3$, we have $G_n=2/9$, whereas $G_n$ without the added $1/n=1/3$ would give the negative value $-1/9$, which is incompatible with the meaning of the index.

Finally, using the same arguments as above but now with the right-most expression for $G_{2}$ given in Sect. 2.2 as our starting point, we arrive at

$$\begin{aligned} G_{2,n}= {\overline{X}\over X_{\lceil n/2 \rceil :n}}-{2\over n^2}\sum _{i=1}^{n} {\sum _{k=1}^{i}X_{k:n} \over X_{\lceil n/2 \rceil :n}} \end{aligned}$$

as an empirical estimator of $G_{2}$. As before, $\overline{X}$ stands for the mean of $X_{1},\dots , X_{n}$.

### An analysis of capital incomes from the ECHP (2001) survey

Using the formulas for calculating the aforementioned indices from data, we now analyze capital incomes, which are income flows from financial assets actually received during the reference year, reported in the European Community Household Panel survey (ECHP 2001) that was conducted by Eurostat in 2001, the last of the eight waves of the survey. In this regard, it is instructive to recall the definition of capital incomes given by T. Piketty:

[C]apital is defined as the sum total of nonhuman assets that can be owned and exchanged on some market. Capital includes all forms of real property (including residential real estate) as well as financial and professional capital (plants, infrastructure, machinery, patents, and so on) used by firms and government agencies. (Piketty 2014, p. 46)

For the importance, especially in the context in Europe, of capital incomes and income transfers, which we later analyze in Sect. 5, we again refer to T. Piketty:

Ultimately, the decline in the capital/income ratio between 1913 and 1950 is the history of Europe’s suicide, and in particular of the euthanasia of European capitalists. (Piketty 2014, p. 149)

Modern redistribution, as exemplified by the social states constructed by the wealthy countries in the twentieth century, is based on a set of fundamental social rights: to education, health, and retirement. Whatever limitations and challenges these systems of taxation and social spending face today, they nevertheless marked an immense step forward in historical terms. Partisan conflict aside, a broad consensus has formed around these social systems, particularly in Europe, which remains deeply attached to what is seen as a “European social model.” (Piketty 2014, p. 481)

For the countries of Europe, the priority now should be to construct a continental political authority capable of reasserting control over patrimonial capitalism and private interests and of advancing the European social model in the twenty-first century. The minor disparities between national social models are of secondary importance in view of the challenges to the very survival of the common European model. (Piketty 2014, p. 561–562)

**Fig. 7:**
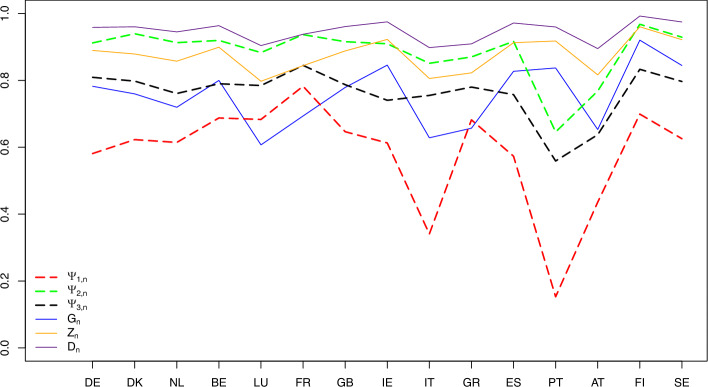
The income-inequality indices $G_{n}$, $Z_{n}$, $D_{n}$, and the indices $\Psi _{k,n}$ for the fifteen European countries with $n=n_P$ specified in Table 3 (based on ECHP 2001)

Specifically, the data come from 59 750 households with 121 122 persons from the fifteen European countries specified in Table 3 using the ISO 3166-1 alpha-2 (two-letter) codes. By looking at the means and medians in Table 3, we see how skewed to the right the distributions of the countries are. Figure 7 (with $G_{2,n}$ excluded due to its large values)visualizes the index values calculated using formulas (4.1)–(4.3) and reported in Table 3. (The arrangement of the countries from left to right is totally arbitrary, and the lines connecting the index values of different countries is only for the purpose of visualization and easier comparison of the countries). For a more detailed description of the data and relevant references, we refer to Greselin et al. (2014, Section 1). Next are several observations based on Table 3 and Fig. 7.

**Table 3:** The income-inequality indices $G_{n}$, $Z_{n}$, $D_{n}$, $G_{2,n}$, and the indices $\Psi _{1,n}$, $\Psi _{2,n}$, $\Psi _{3,n}$ for the fifteen European countries with $n=n_P$, where $n_P$ is the number of people in the sample who possess capital incomes, and $n_T$ is the total sample size of the given country (based on ECHP 2001)

Countries	Means	Medians	Sample sizes		Inequality indices							Ranks based on		
			$n_{T}~~$	$n_{P}~~$	$G_{n}$	$Z_{n}$	$D_{n}$	$G_{2,n}$	$\Psi _{1,n}$	$\Psi _{2,n}$	$\Psi _{3,n}$	$\Psi _{1,n}$	$\Psi _{2,n}$	$\Psi _{3,n}$
DE	948.373	186.622	10,624	4861	0.782	0.890	0.959	3.975	0.581	0.912	0.809	5	7	13
DK	1071.062	231.417	3789	1135	0.760	0.879	0.961	3.512	0.623	0.940	0.798	8	14	12
NL	660.744	214.184	8608	2863	0.720	0.858	0.945	2.219	0.615	0.913	0.761	7	8	6
BE	5309.168	1374.805	4299	690	0.800	0.899	0.964	3.091	0.688	0.920	0.790	13	11	10
LU	1982.621	1214.678	4916	769	0.607	0.798	0.904	0.989	0.683	0.883	0.785	12	5	8
FR	716.679	359.932	10,119	4347	0.694	0.844	0.938	1.381	0.783	0.937	0.845	15	13	15
GB	1522.177	368.826	8521	3477	0.779	0.888	0.961	3.214	0.647	0.916	0.787	10	9	9
IE	604.580	99.040	4023	949	0.846	0.923	0.975	5.157	0.613	0.910	0.741	6	6	3
IT	1.762	0.480	13,392	1111	0.628	0.806	0.898	2.303	0.341	0.851	0.755	2	3	4
GR	2256.554	1232.575	9419	335	0.657	0.823	0.909	1.197	0.682	0.870	0.780	11	4	7
ES	240.838	37.431	11,964	6541	0.827	0.913	0.972	5.322	0.573	0.917	0.758	4	10	5
PT	1232.674	116.260	10,915	600	0.837	0.918	0.960	8.862	0.153	0.646	0.559	1	1	1
AT	323.822	133.500	5605	2834	0.653	0.817	0.895	1.585	0.436	0.768	0.638	3	2	2
FI	3662.567	180.634	5637	1509	0.921	0.961	0.993	18.651	0.699	0.968	0.833	14	15	14
SE	601.528	84.495	9291	5637	0.845	0.922	0.975	6.013	0.626	0.929	0.797	9	12	11

Portugal has the lowest value of $\Psi _{1,n}$, with the median income of the poorest $p \times 100 \%$ persons equal, after averaging over all $p \in (0,1)$, to $84.7\%$ of the median income of the entire population.

The opposite happens in France, which provides the highest contrast among the countries when comparing the median income of the poorest $p\times 100 \%$ persons with the overall median income: after averaging such ratios over all $p \in (0,1)$, we obtain $21.7\%$.

For France, we also observe the largest value of $\Psi _{3,n}$. The median income of the poorest $p \times 100 \%$ people is equal, after averaging over all $p \in (0,1)$, to only $15.5\%$ of the median income of the richest $p \times 100 \%$ persons in the population.

When we are interested in comparing the median income of the poorest $p\times 100 \%$ persons with the median income of the remaining $(1-p)\times 100\%$ part of the population, the index $\Psi _{2,n}$ tells us that Finland is the country in which such a contrast, after averaging over all $p \in (0,1)$, is the largest.

**Fig. 8:**
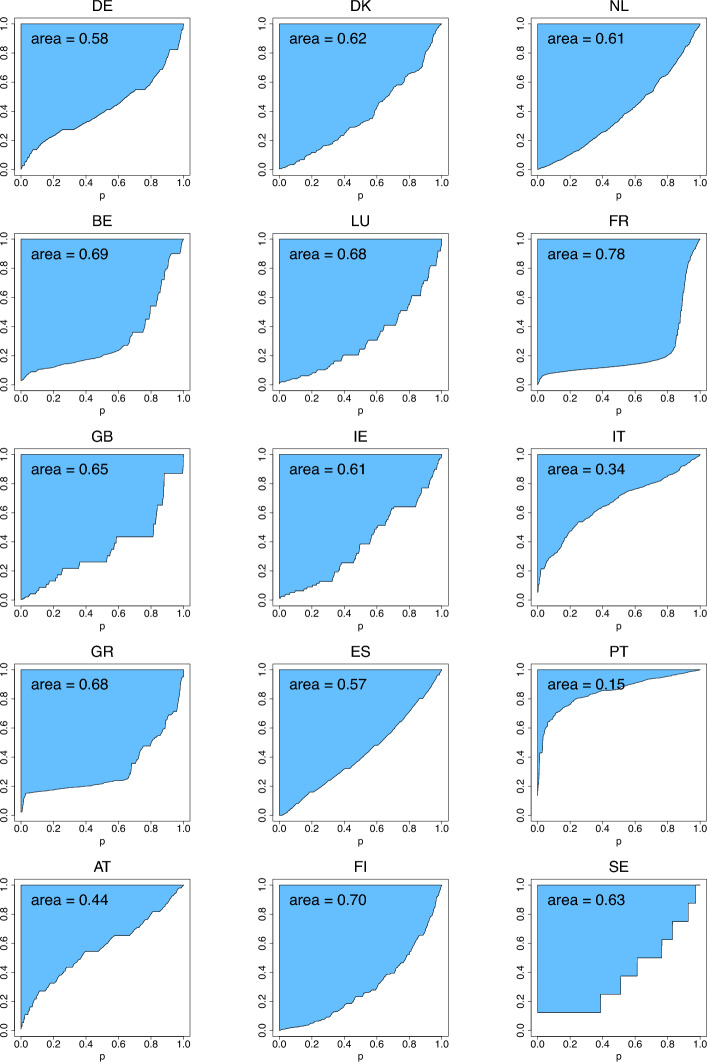
The income-equality curve $\psi _{1,n}$ and the shaded-in area (i.e., $\Psi _{1,n}$) above it for the fifteen European countries, where $n=n_P$ is specified in Table 3 (based on ECHP 2001)

**Fig. 9:**
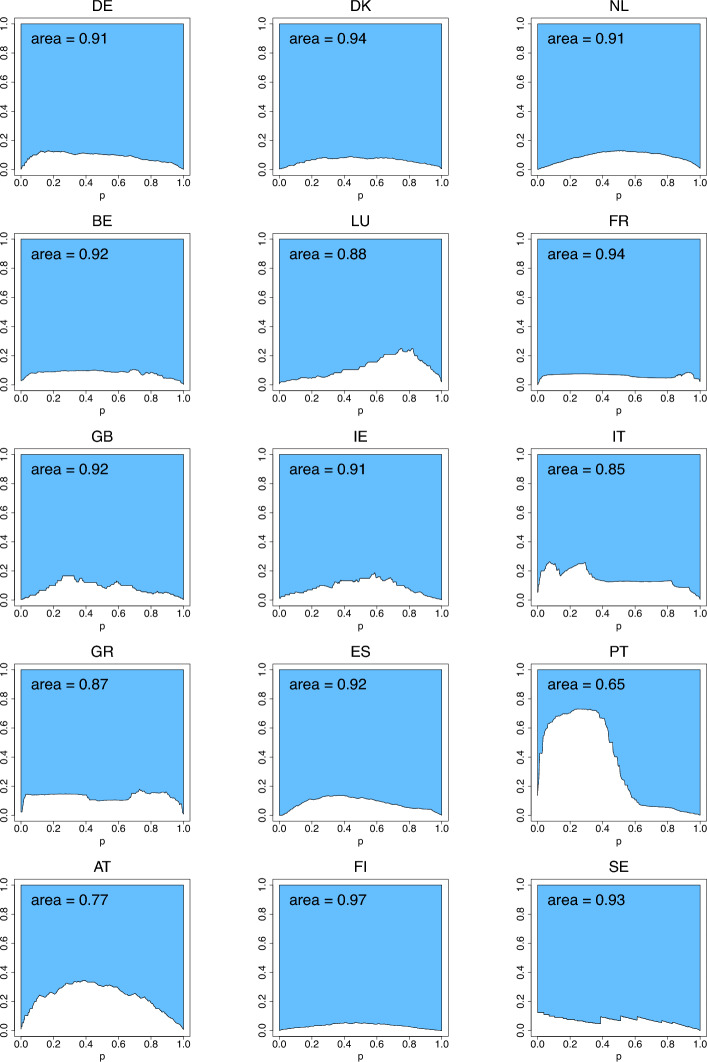
The income-equality curve $\psi _{2,n}$ and the shaded-in area (i.e., $\Psi _{2,n}$) above it for the fifteen European countries, where $n=n_P$ is specified in Table 3 (based on ECHP 2001)

**Fig. 10:**
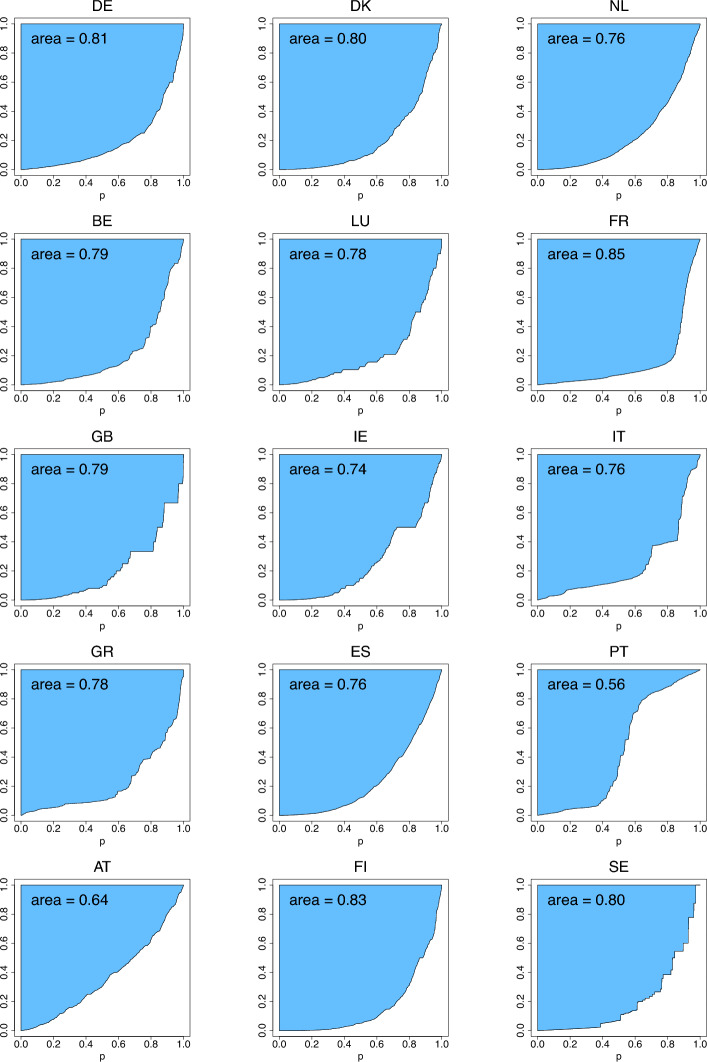
The income-equality curve $\psi _{3,n}$ and the shaded-in area (i.e., $\Psi _{3,n}$) above it for the fifteen European countries, where $n=n_P$ is specified in Table 3 (based on ECHP 2001)

Figures 8, 9, 10depict the three income-equality curves $\psi _{k,n}$ for the fifteen European countries specified in Table 3, with the shaded-in areas above them depicting the values of the indices $\Psi _{k,n}$. The curves have been obtained via formulas (2.3)–(2.7) by replacing *Q* by $Q_n$ given by Eq. (4.4) with $n=n_P$, where $n_P$ is the number of people in the sample who possess capital incomes, and $n_T$ is the total sample size of the given country.

Comparing the plots of Figs. 8, 9, 10 derived from the actual data with the ones of Figs. 4, 9, 6 generated from the parametric distributions, for most of the countries we see that the distributions of capital incomes are right-skewed and similar to most of our illustrative choices (e.g., gamma, lognormal, Pareto, Weibull). To reach a more definitive answer, formal statistical analysis should be performed using the methods provided by Prendergast and Staudte (2016).

### A comparison with capital incomes from the EU-SILC (2018) survey

To get an insight into more recent European situation, we further analyse data coming from the EU Statistics on Income and Living Conditions survey (EU-SILC 2018), which substituted the ECHP survey after its eighth wave in 2001.

We note at the outset that in the EU-SILC survey, the capital incomes are available only at the level of households, and sample sizes are approximately seven times larger if compared with the earlier ECHP survey. Hence, the EU-SILC data give rise to more accurate estimates. In our study we use the following variables:

HY040G: income from rental of a property or land.
HY090G: interests, dividends, profit from capital investments in unincorporated business.
PY080: pensions received from individual private plans.

Namely, for each household we sum up HY040G and HY090G, and then add all the pensions received by a component of the same household (variable PY080). This gives us the capital incomes for each household. Denote them by, say, $Z_1,\dots , Z_n$. As the data refer to households, an equivalence scale needs to be employed to make meaningful comparisons of monetary incomes of social units with different numbers of inhabitants, and to also take into account the economies of scale (within each household) with regard to the consumption of certain goods. An equivalence scale acts as a weight, giving rise to an *equivalence income* that can be used for inequality, poverty and welfare analyses. We opt for the “square root” equivalence scale, adopted by the Organization for Economic Cooperation and Development (OECD) in their recent publications. Namely, each household income $Z_i$ is divided by the square root of the household size $w_i$, yielding $X_i$, $i=1,\ldots ,n$, that we use in our analysis.

We analyse the same fifteen European countries as in previous Sect. 4.3, and consider the 340 540 households surveyed by the EU-SILC in 2018. A summary is provided in Table 4.For a useful comparison of means and medians, we apply the official average national currency exchange rates (year 2018) for the three countries that have not adopted the Euro: Denmark, Great Britain, and Sweden, whose currencies are the Danish Krone, the British Pound, and the Swedish Krona, respectively. Hence, all the analyzed data are in Euro.

**Table 4:** The income-inequality indices $G_{n}$, $Z_{n}$, $D_{n}$, $G_{2,n}$, and the indices $\Psi _{1,n}$, $\Psi _{2,n}$, $\Psi _{3,n}$ for the fifteen European countries with $n=n_P$, where $n_P$ is the number of people in the sample who possess capital incomes, and $n_T$ is the total sample size of the given country (based on EU-SILC 2018)

Countries	Means	Medians	Sample sizes		Inequality indices							Ranks based on		
			$n_{T}~~$	$n_{P}~~$	$G_{n}$	$Z_{n}$	$D_{n}$	$G_{2,n}$	$\Psi _{1,n}$	$\Psi _{2,n}$	$\Psi _{3,n}$	$\Psi _{1,n}$	$\Psi _{2,n}$	$\Psi _{3,n}$
DE	1515.759	147.000	25,784	20,332	0.845	0.922	0.980	8.711	0.549	0.940	0.762	3	4	3
DK	691.532	83.539	16,812	5118	0.846	0.923	0.983	7.003	0.746	0.978	0.858	12	10	9
NL	1542.372	85.333	24,986	19,192	0.914	0.957	0.991	16.521	0.644	0.955	0.806	6	5	5
BE	1833.003	54.286	11,892	6568	0.873	0.936	0.990	29.860	0.699	0.962	0.881	9	6	13
LU	3193.057	124.500	7666	4400	0.853	0.927	0.988	21.883	0.627	0.972	0.822	5	9	7
FR	4300.964	453.333	21,752	17,828	0.848	0.924	0.984	8.048	0.745	0.979	0.862	11	11	10
GB	2811.430	442.439	34,226	15,090	0.788	0.894	0.971	5.010	0.695	0.963	0.842	8	8	8
IE	4653.139	1080.000	8764	1678	0.754	0.877	0.968	3.245	0.823	0.983	0.891	15	13	14
IT	2004.340	266.667	42,346	22,188	0.808	0.904	0.976	6.075	0.663	0.963	0.822	7	7	6
GR	3216.821	1966.815	48,610	7512	0.579	0.781	0.892	0.947	0.598	0.866	0.710	4	1	2
ES	2132.438	264.200	26,736	13,246	0.806	0.903	0.978	6.504	0.739	0.980	0.865	10	12	11
PT	2266.447	694.447	27,434	5516	0.703	0.849	0.941	2.292	0.528	0.910	0.705	1	2	1
AT	1699.386	103.740	12,206	8598	0.877	0.938	0.987	14.358	0.543	0.934	0.765	2	3	4
FI	3525.164	203.167	19,664	16,008	0.854	0.927	0.988	14.831	0.809	0.993	0.900	14	15	15
SE	312.698	33.170	11,662	9138	0.836	0.918	0.983	7.880	0.761	0.985	0.868	13	14	12

**Fig. 11:**
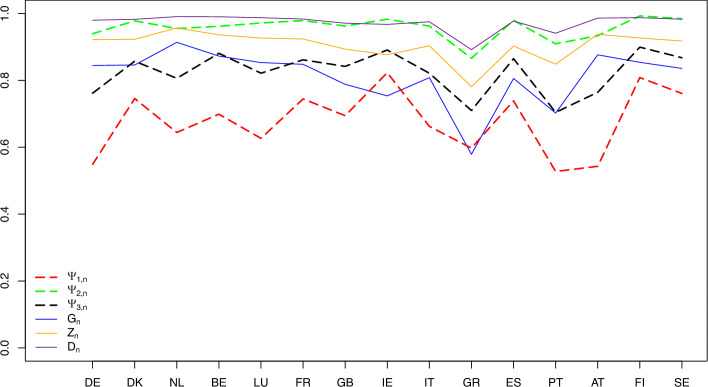
The income-inequality indices $G_{n}$, $Z_{n}$, $D_{n}$, and the indices $\Psi _{k,n}$ for the fifteen European countries with $n=n_P$ specified in Table 4 (based on EU-SILC 2018)

**Fig. 12:**
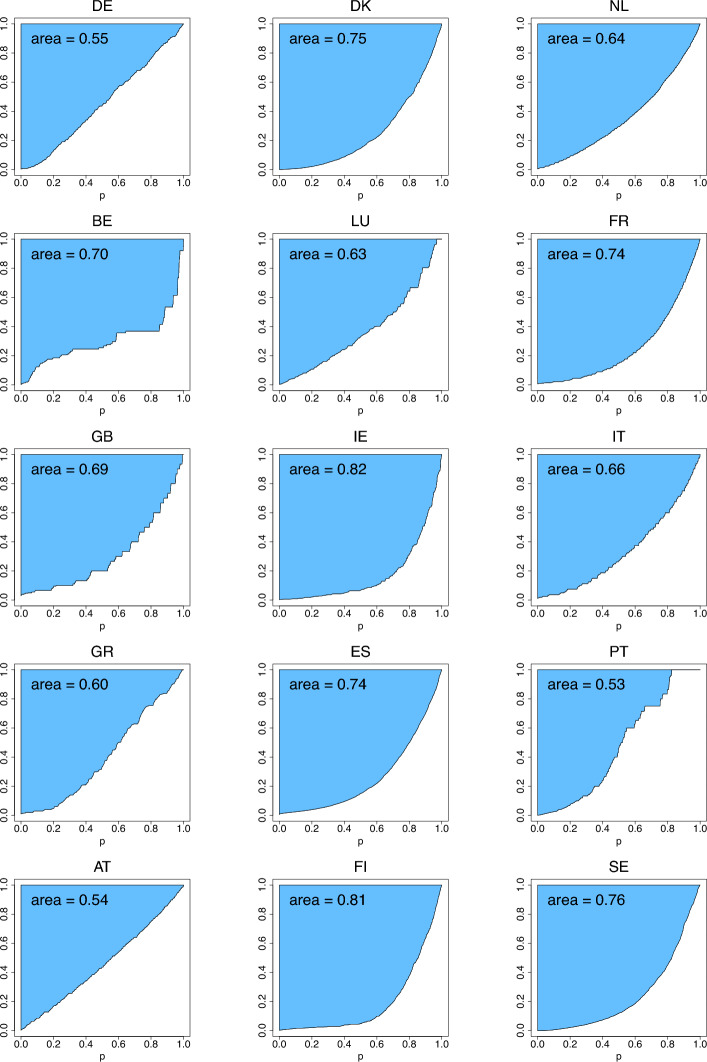
The income-equality curve $\psi _{1,n}$ and the shaded-in area (i.e., $\Psi _{1,n}$) above it for the fifteen European countries, where $n=n_P$ is specified in Table 4 (based on EU-SILC 2018)

**Fig. 13:**
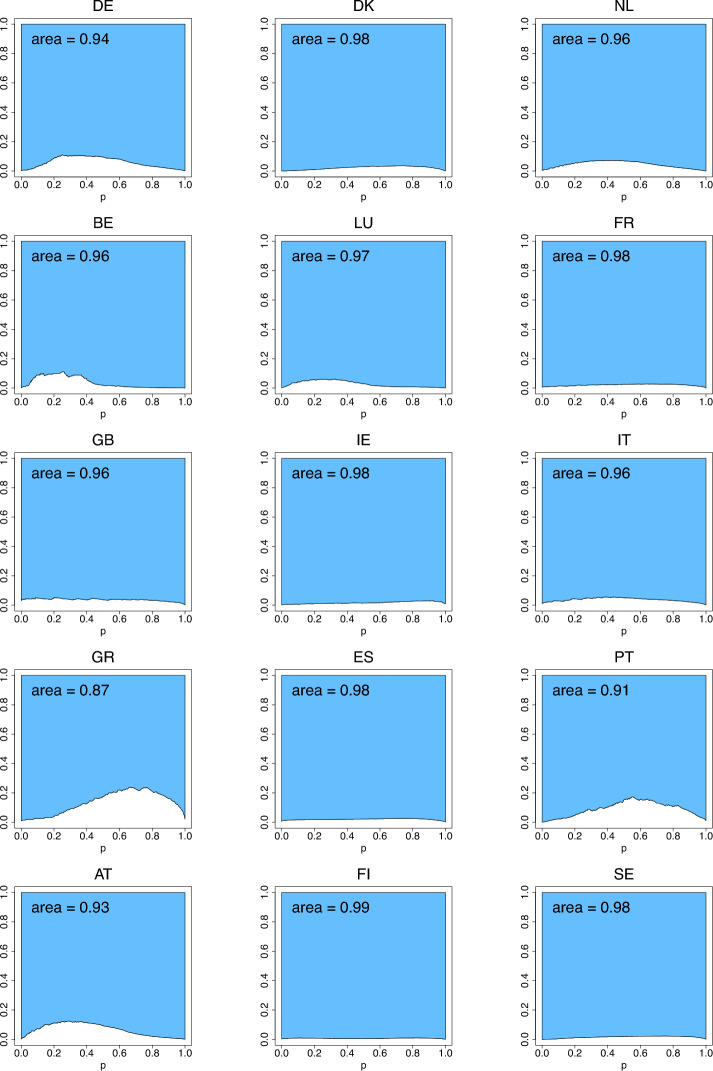
The income-equality curve $\psi _{2,n}$ and the shaded-in area (i.e., $\Psi _{2,n}$) above it for the fifteen European countries, where $n=n_P$ is specified in Table 4 (based on EU-SILC 2018)

**Fig. 14:**
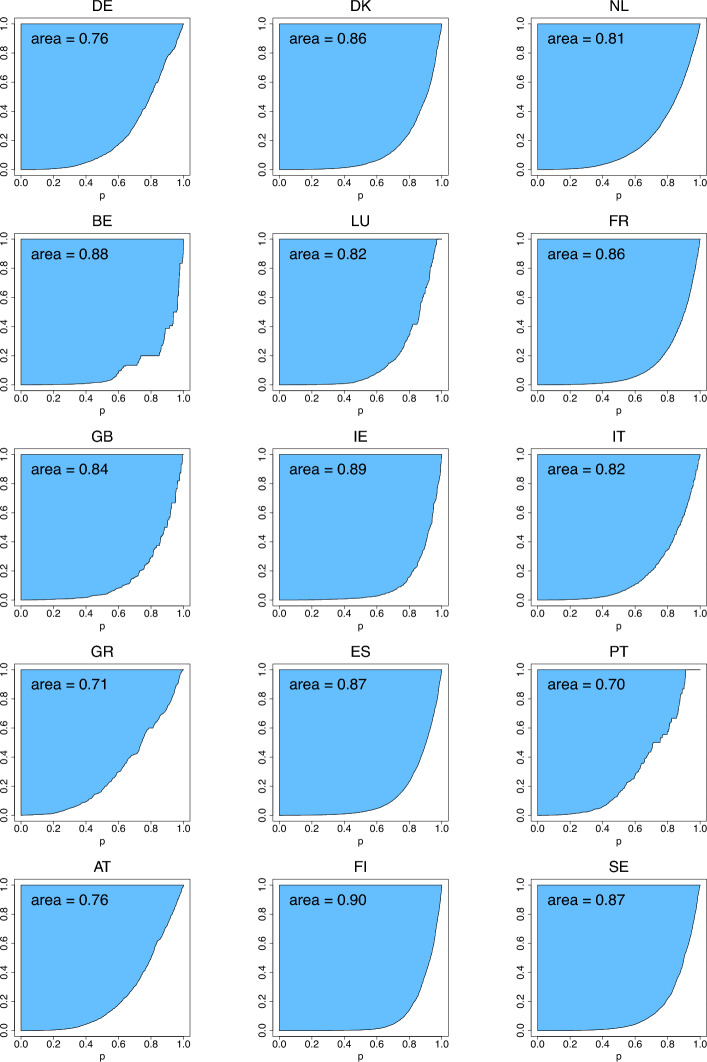
The income-equality curve $\psi _{3,n}$ and the shaded-in area (i.e., $\Psi _{3,n}$) above it for the fifteen European countries, where $n=n_P$ is specified in Table 4 (based on EU-SILC 2018)

The differences between the means and medians in Table 4 facilitate the assessment of skewness of income distributions. The list of countries with lower inequality (having a two-digit rank in at least one of the indices) is comprised of Denmark, Benelux, France, Ireland, Spain, Finland and Sweden. To compare with the 2001 data, Ireland has joined the list while Germany, Luxembourg, Great Britain and Greece left it. Portugal, that was the country with the highest inequality in 2001, in 2018 was joined by Greece in the list for the primacy of the highest inequality, as seen from the rankings produced by the three indices. Fig. 11 (with $G_{2,n}$ excluded due to its large values) visualizes the index values calculated using formulas (4.1)–(4.3) and reported in Table 4. As in the case of Fig. 7, the arrangement of the countries from left to right in Fig. 11 is arbitrary, although follows exactly that of Fig. 7, and the lines connecting the index values of different countries is only for the purpose of visual comparison of the countries. Figs. 12, 13, 14 depict the three income-equality curves $\psi _{k,n}$ for the fifteen European countries specified in Table 4, with the shaded-in areas above them depicting the values of the indices $\Psi _{k,n}$.

## The effects of income transfers on the indices $\Psi _{k,n}$

We have already alluded to the importance of income redistribution in Sect. 4.3, with a number of quotes on the subject from Piketty (2014). In the current section, we present a mathematical treatment of income transfers in terms of the data-driven versions $\Psi _{k,n}$ of the income inequality indices $\Psi _k$, $k=1,2,3$.

Consider *n* persons whose ordered incomes we denote by $X_{1:n}< \cdots < X_{n:n}$. Choose any pair from these persons and call them *L* and *H*. The person $L \in \{1,\dots , n-1\}$ possesses income $X_{L:n}$ and the person $H \in \{2,\dots , n\}$ possesses income $X_{H:n}$. We assume $L<H$. Hence, *L* has less income than *H*, that is, $X_{L:n}< X_{H:n}$. Denote $\textbf{X}=(X_{1:n}, \dots , X_{n:n})$.

Assume now that *H* transfers a positive amount $c>0$ to *L* without changing the income ordering among the *n* persons. The transfer produces $\textbf{X}'=(X_{1:n}', \dots , X_{n:n}')$ with the same ordering $X_{1:n}'< \cdots < X_{n:n}'$ of the coordinates as in the case of $\textbf{X}$. (See Appendix 1 for additional technical details). Succinctly, we denote the transfer by

5.1 $$\begin{aligned} L {\mathop {\longleftarrow }\limits ^{c}} H \end{aligned}$$

and read it, e.g., “*L* receives amount *c* from *H*” or “*H* transfers amount *c* to *L*.” We are interested in how the three indices $\Psi _{k,n}=\Psi _{k,n}(\textbf{X})$ react to such transfers, that is, when $\textbf{X}$ turns into $\textbf{X}'$.

**Fig. 15:**
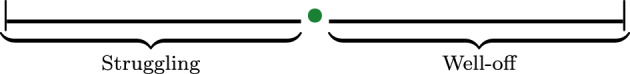
The median (green) delineates the struggling group from the well-off. (Color figure online)

In addition to *L* and *H*, we also involve the “median” person

$$\begin{aligned} M:=\lceil n/2 \rceil \end{aligned}$$

whose income is $X_{M:n}=Q_n(1/2)$ as per Eq. (4.4) with $p=1/2$. Any person *P* with income above the median (i.e., when $P>M$) is called *well-off*, and any person *P* with income below the median (i.e., when $P<M$) is called *struggling* (see Fig. 15).In what follows, we shall be interested in the effects of transfer (5.1) on the three indices when both *L* and *H* are well-off, both are struggling, and when one of them (i.e., *L*) is struggling and the other one (i.e., *H*) is well-off.

Before going into details, we note that the classical Pigou-Dalton principle (PDP) – when it holds – says that $\Psi _{k,n}(\textbf{X})\ge \Psi _{k,n}(\textbf{X}')$ in its weak form and $\Psi _{k,n}(\textbf{X})> \Psi _{k,n}(\textbf{X}')$ in its strong form. As we shall soon see, the three indices will tell us a richer story. Based on it, we shall be able to choose a preferred index, or at least be prompted to think outside the box, which is necessary as Amiel and Cowell (1999) have convincingly argued.

### Index $\Psi _{1,n}$

#### Property 4

In the case of struggling *L* and well-off *H* (i.e., $L<M<H$), the transfer $L {\mathop {\longleftarrow }\limits ^{c}} H$ decreases the value of the index $\Psi _{1,n}$, that is, we have $\Psi _{1,n}(\textbf{X})> \Psi _{1,n}(\textbf{X}')$.

#### Property 5

When both *L* and *H* are well-off (i.e., $M<L<H$), or when both are struggling (i.e., $L<H< M$), the transfer $L {\mathop {\longleftarrow }\limits ^{c}} H$ does not change the value of the index $\Psi _{1,n}$, that is, we have $\Psi _{1,n}(\textbf{X})= \Psi _{1,n}(\textbf{X}')$.

These two properties say that in order to decrease income inequality based on the index $\Psi _{1,n}$, a well-off person needs to transfer some amount to a struggling person, whereas any transfer between two well-off persons or between two struggling ones does not make any difference.

### Index $\Psi _{2,n}$

The index $\Psi _{2,n}$ is more sensitive to transfers than the previous index. Specifically, we shall see from the following properties that $\Psi _{2,n}$ decreases when $L {\mathop {\longleftarrow }\limits ^{c}} H$, unless both *H* and *L* are well-off and *H* transfers to *L* only a small amount $c>0$.

#### Property 6

In the case of struggling *L* and well-off *H* (i.e., $L< M < H$), or when both *L* and *H* are struggling (i.e., $L<H< M$), the transfer $L {\mathop {\longleftarrow }\limits ^{c}} H$ decreases the value of the index $\Psi _{2,n}$, that is, $\Psi _{2,n}(\textbf{X})> \Psi _{2,n}(\textbf{X}')$.

#### Property 7

When both *L* and *H* are well-off (i.e., $M<L<H$), the transfer $L {\mathop {\longleftarrow }\limits ^{c}} H$ implies $\Psi _{2,n}(\textbf{X})> \Psi _{2,n}(\textbf{X}')$ when

5.2 $$\begin{aligned} c> c_2:={X_{L-M:n}X^2_{H:n} - X_{H-M:n}X^2_{L:n} \over X_{L-M:n}X_{H:n} + X_{H-M:n}X_{L:n} }. \end{aligned}$$

Furthermore, we have $\Psi _{2,n}(\textbf{X})= \Psi _{2,n}(\textbf{X}')$ in the “boundary” case $c=c_2$, and $\Psi _{2,n}(\textbf{X})< \Psi _{2,n}(\textbf{X}')$ when $c<c_2$.

Hence, the index $\Psi _{2,n}$ avoids giving the impression of inequality reduction when only a small amount is transferred among well-off persons. In other words, for the index to decrease in the case of two well-off persons, the richer one needs to transfer a sufficiently large amount in order to qualify for inequality reduction. Next is an example illustrating Properties 6 and 7.

#### Example 2

Consider a group of seven persons, among whom there are three struggling ones (denoted by *S*’s) and three well-off persons (denoted by *W*’s). The person *M* has the median income $X_{M:7}$ among these seven persons, and thus a “7” in its notation. Let their incomes be

5.3 $$\begin{aligned} \textbf{X}&=(X_{1:7},X_{2:7},X_{3:7},X_{4:7},X_{5:7},X_{6:7},X_{7:7}) \nonumber \\&=(X_{S_1:7},X_{S_2:7},X_{S_3:7},X_{M:7},X_{W_1:7},X_{W_2:7},X_{W_3:7}) \nonumber \\&= (\underbrace{1, 3, 5,}_{\text {Incomes of } S'\text {s}} \overbrace{7,}^{\text {Income of } M} \underbrace{10, 20, 24}_{\text {Incomes of } W'\text {s}}) . \end{aligned}$$

**Fig. 16:**
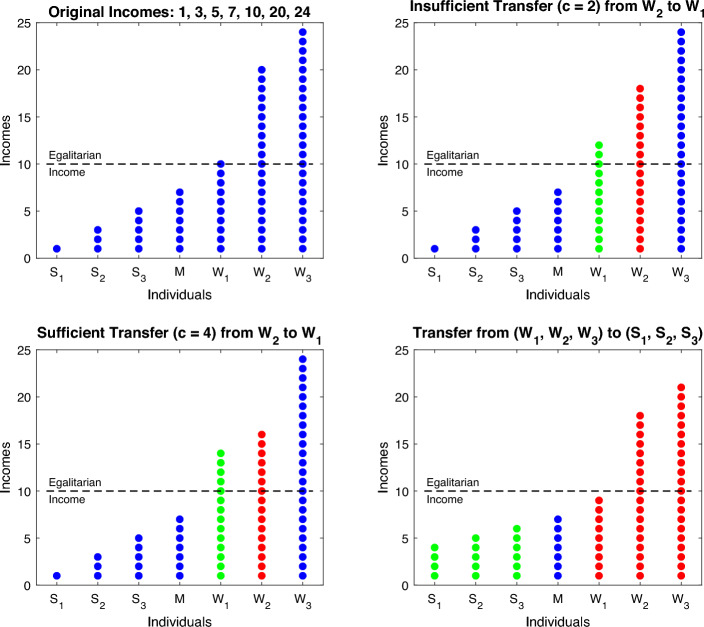
Distributions of incomes with dots representing units, or amounts, of income: the blue dots correspond to the original distribution of incomes, the red ones correspond to reduced incomes due to transfers, and the green dots correspond to increased incomes. (Color figure online)

The index of inequality for this vector is $\Psi _{2,n}=0.8472$. Hence, $n=7$ and thus $M=\lceil 3.5 \rceil =4$, which gives the median income $X_{4:7}=7$. There are three struggling persons $S_1$, $S_2$, and $S_3$ with incomes 1, 3, and 5, respectively, and three well-off persons $W_1$, $W_2$, and $W_3$ with incomes 10, 20, and 24, respectively (see the top-left panel in Fig. 16 for a visualization). The horizontal dashed line in each panel of Fig. 16, noted as “egalitarian income” and plotted at the height 10, refers to the egalitarian redistribution of the above specified incomes (whose sum is equal to 70) among the seven participating persons. Various transfers of the incomes are visualized in the other panels of the figure, with red dots depicting the incomes of the person(s) whose incomes decreased due to transfers to less fortunate one(s), highlighted in green. We next discuss what we see in the three panels of Fig. 16 depicting various transfers.

*Top-right panel.*

The panel depicts the transfer $W_1{\mathop {\longleftarrow }\limits ^{c}}W_2$ among two well-off persons of the insufficient for inequality decrease amount $c=2$. Hence, the resulting distribution

5.4 $$\begin{aligned} (1, 3, 5, 7, 12, 18, 24) \end{aligned}$$

retains the same value of the index $\Psi _{2,n}=0.8472$ as distribution (5.3).

To see what amounts are sufficient and what insufficient, we note that condition (5.2) is equivalent to

$$\begin{aligned} c>c_2= {1\times 20^2 - 3\times 10^2 \over 1\times 20 + 3\times 10 } = 2. \end{aligned}$$

For the ordering of incomes to remain the same after the transfer $L {\mathop {\longleftarrow }\limits ^{c}} H$, we need the restriction

$$\begin{aligned} c< {X_{H:7}-X_{L:7} \over 2}=5. \end{aligned}$$

Hence, to decrease income inequality according to the index $\Psi _{2,n}$, the person *H* needs to transfer to *L* more than 2, but less than 5 to avoid swapping the position with *L*.

*Bottom-left panel.*

The panel depicts the transfer $W_1{\mathop {\longleftarrow }\limits ^{c}}W_2$ of the sufficient for inequality decrease amount $c=4$, in which case we have

5.5 $$\begin{aligned} (1, 3, 5, 7, 14, 16, 24) \end{aligned}$$

with the value of the index $\Psi _{2,n}=0.8442$.

*Bottom-right panel.*

We now consider a more complex situation when every well-off person commits to improving the incomes of the three struggling persons, with the final distribution of incomes becoming (4, 5, 6, 7, 9, 18, 21) . We can achieve this distribution in several steps, each reducing income inequality and maintaining the original ordering of the seven persons. Recall that we start from the vector (1, 3, 5, 7, 10, 20, 24) , whose inequality index is $\Psi _{2,n}=0.8472$, and the steps could be these: The transfer $S_3 {\mathop {\longleftarrow }\limits ^{1}} W_1$ results in the distribution

5.6 $$\begin{aligned} (1, 3, 6, 7, 9, 20, 24) \end{aligned}$$

with the index $\Psi _{2,n}=0.8296$. The transfer $S_2{\mathop {\longleftarrow }\limits ^{2}} W_2$ results in

5.7 $$\begin{aligned} ( 1, 5, 6, 7, 9, 18, 24) \end{aligned}$$

with the index $\Psi _{2,n}=0.7870$. Finally, the transfer $S_1{\mathop {\longleftarrow }\limits ^{3}} W_3$ results in the distribution

5.8 $$\begin{aligned} (4, 5, 6, 7, 9, 18, 21) \end{aligned}$$

depicted in the bottom right panel of Fig. 16 and having the index $\Psi _{2,n}=0.6640$. All these are inequality-reducing transfers from well-off persons to struggling ones. A continuation of this example is given in Appendix 1 with another instructive set of steps leading to distribution (5.8).

### Index $\Psi _{3,n}$

#### Property 8

In the case of struggling *L* and well-off *H* (i.e., $L<M<H$), the transfer $L {\mathop {\longleftarrow }\limits ^{c}} H$ decreases the value of the index $\Psi _{3,n}$, that is, $\Psi _{3,n}(\textbf{X})> \Psi _{3,n}(\textbf{X}')$.

#### Property 9

When both *L* and *H* are well-off (i.e., $M<L<H$), or when both are struggling (i.e., $L<H< M$), the transfer $L {\mathop {\longleftarrow }\limits ^{c}} H$ increases the value of the index $\Psi _{3,n}$, that is, we have $\Psi _{3,n}(\textbf{X})< \Psi _{3,n}(\textbf{X}')$.

Hence, when the goal is to decrease income inequality, these two properties say that well-off persons must transfer to struggling persons, and the index discourages transfers between two well-off persons, or between two struggling ones, as the index views such transfers manipulative with no real consequences. Whether we agree with this or not determines whether or not we want adopt the index $\Psi _{3,n}$ for measuring income inequality.

### A numerical example

Having by now discussed the three indices and their properties, we next have a numerical example that illustrates the performance of the three indices side-by-side. Namely, consider the six distributions of incomes specified in (5.3)–(5.8) and visualized in Fig. 16. Table 5 contains the numerical values of the three indices for the six income distributions. Note that the original (or initial) incomes are given by distribution (5.3), from which various transfers are executed, with post-transfer index values given in the columns to the right of (5.3). We next discuss these post-transfer index values with respect to the pre-transfer values, which are reported in column (5.3). For this, it is instructive to have the six distributions side-by-side, with the median (equal to 7) in bold separating the struggling (on the left) from the well-off (on the right) persons:

$$\begin{aligned}&(5.3): (1, 3, 5, {\textbf {7}}, 10, 20, 24) \\&(5.4): (1, 3, 5, {\textbf {7}}, 12, 18, 24) \\&(5.5): (1, 3, 5, {\textbf {7}}, 14, 16, 24) \\&(5.6): (1, 3, 6, {\textbf {7}}, 9, 20, 24) \\&(5.7): (1, 5, 6, {\textbf {7}}, 9, 18, 24) \\&(5.8): (4, 5, 6, {\textbf {7}}, 9, 18, 21) \end{aligned}$$

The index $\Psi _{1,n}$ values remain unchanged after the transfers from (5.3) to (5.4), and also from (5.3) to (5.5), because the transfers are among the well-off persons (Property 5). The index values decrease more and more when the transfers from (5.3) are made to (5.6), (5.7), and (5.5), because the three transfers are from well-off persons to the struggling ones (Property 4), and more and more are being transferred to the struggling persons, as seen by comparing distributions (5.6)–(5.8).

**Table 5:** The three indices for income distributions (5.3)–(5.8)

Indices	(5.3)	(5.4)	(5.5)	(5.6)	(5.7)	(5.8)
$\Psi _{1,n}$	0.5714	0.5714	0.5714	0.5238	0.4286	0.2857
$\Psi _{2,n}$	0.8472	0.8472	0.8442	0.8296	0.7870	0.6640
$\Psi _{3,n}$	0.7694	0.7917	0.8046	0.7139	0.6713	0.6217

In minute details, the performance of the index $\Psi _{2,n}$ has been discussed in Example 2. Here is its summary: The value of $\Psi _{2,n}$ does not change when moving from distribution (5.3) to (5.4) because the transfer amount is the boundary case (Property 7), meaning that it neither increases nor decreases the index. The transfer from (5.3) to (5.5) is, however, sufficiently large to decrease the index, even though the transfer occurs among the well-off persons (Property 7). The transfers from (5.3) to (5.6), (5.7), and (5.5) are from well-off persons to struggling ones, and since increasing amounts are being transferred, the index $\Psi _{2,n}$ values decrease more and more (Property 6).

Contrary to what the classical Pigou-Dalton principle postulates, the index $\Psi _{3,n}$ discourages transfers among the well-off (as well as among the struggling) persons (Property 9). This is reflected by the increased values of the index in the case of transfers from (5.3) to (5.4), and also from (5.3) to (5.5). The index, however, starts to decrease, and more so, for transfers from (5.3) to (5.6), (5.7), and (5.5), because the three transfers are from well-off persons to the struggling ones (Property 8).

## Conclusion

In this paper we have explored three inequality indices that reflect three different views of measuring income inequality:

1. The median income of the poor is compared with the median income of the entire population. This is index $\Psi _{1,n}$. It decreases when a well-off person transfers any amount to a struggling one, provided that the transfer does not change the ranking of the persons. However, the index does not change when the transfer happens between two well-off persons, or between two struggling ones, provided that the transfer does not change the ranking of the persons.
2. The median income of the poor is compared with the median income of those who are not poor. This is index $\Psi _{2,n}$. It decreases when a well-off person transfers any amount to a struggling one, or when transfer occurs among two struggling persons, provided that the transfer does not change the ranking of the persons. However, only large transfers among well-off persons decrease the index, and increase when only small amounts are transferred, provided that the transfers do not change the ranking of the persons.
3. The median income of the poor is compared with the median of the same proportion of the richest. This is index $\Psi _{3,n}$. It decreases when a well-off person transfers any amount to a struggling one, provided that the transfer does not change the ranking of the persons. The index, however, increases when two well-off persons transfer any amount among themselves, or when struggling persons transfer any amount among themselves, provided that the transfer does not change the ranking of the persons.

Hence, in view of how transfers affect the indices, we may decide which of the three indices to use (or not to use) in actual data analyses. For example, when it is of interest to see whether well-off persons help the poor ones, irrespective of what is happening inside the well-off group, or inside the struggling one, then the index $\Psi _{1,n}$ should be preferred.

To facilitate practical implementation and analyses at the data and population levels, we have presented the three inequality indices and their equality curves in two ways: one that is suitable for modeling populations, and the other one that is suitable for direct data-focused computations. In particular, the indices and their curves have been illustrated using popular parametric models of income distributions, and also calculated and interpreted using real data. Such results facilitate the development of statistical inference, as seen from the contributions by Prendergast and Staudte (2016, 2018), Oancea and Pirjol (2019), Jokiel-Rokita and Pia̧tek (2023), and Pia̧tek (2023).

Important statistical work remains to be done in the area. For example, decomposition of the indices by subpopulations, income components, intra- and inter-groups inequalities are among the topics of immediate interest (e.g, Amate-Fortes et al. 2021; Qiu et al. 2021), and for a sample of methodological research in the case of the Gini, Zenga, and related indices we refer to Radaelli (2010), Porro and Zenga (2020), and Zenga and Jȩdrzejczak (2020).

The indices do not require any finite moment and therefore are suitable to analyze any population, including ultra heavily tailed, i.e., without any finite moment, unlike the Gini and many other classical indices whose definitions require a finite first moment. Developing statistical inference in such situations usually relies on Extreme Value Theory, and for a glimpse of related to our current study research, we refer to Greselin et al. (2014), where, based on empirical evidence, it is noted that some income distributions may not have finite first moments.

## A Technicalities

### Proof

(Justification of definitions (4.1)–(4.3)) The three empirical indices arise from formulas (2.4)–(2.8) by first replacing the population quantile function *Q* by the empirical quantile function $Q_n$ in all the formulas. (We have asymptotically insignificantly modified the obtained expressions to facilitate their intuitive appeal). In detail, with $F_n$ denoting the empirical cumulative distribution function based on $X_{1},\dots , X_{n}$, the empirical quantile function is given by Eq. (4.4). Thus, for example, $Q_n(1/2)=X_{M:n}$ with $M=\lceil n/2 \rceil$ is the empirical median used in the definition of $\Psi _{1,n}$. Note also that $\lfloor n/2 \rfloor + M = n$, and thus the definition of the index $\Psi _{2,n}$ does not go beyond the random variables $X_{1},\dots , X_{n}$. $\square$

### Proof of Property 2

The inequality holds because $a/b\le (a+c)/(b+c)$ for all (positive) $a\le b$ and $c\ge 0$, and to have the strict inequality, we note that $a/b< (a+c)/(b+c)$ holds for all (positive) $a< b$ and $c> 0$.

### Proof of Property 3

The property follows from $(a+c)/(b+c)\rightarrow 1$ when $c\rightarrow \infty$ irrespective of the values of (positive) $a\le b$.

### Proof

(Details of definition (5.1))

$$\begin{aligned} X_{i:n}'&=X_{i:n} \quad \text {for} \quad 1\le i \le L-1,\\ X_{L:n}'&=X_{L:n}+c ,\\ X_{i:n}'&=X_{i:n} \quad \text {for} \quad L+1\le i \le H-1,\\ X_{H:n}'&=X_{H:n}-c ,\\ X_{i:n}'&=X_{i:n} \quad \text {for} \quad H+1\le i \le n, \end{aligned}$$

where *L* and *H* are integers such that $1\le L<H\le n$, and $c>0$ is any positive real number (i.e., the amount transferred from *H* to *L*) such that the following ordering holds:

6.1 $$\begin{aligned} X_{1:n}< \cdots< X_{L-1:n}< X_{L:n}+c< X_{L+1:n}< \cdots< X_{H-1:n}< X_{H:n}-c< X_{H+1:n}< \cdots < X_{n:n}. \end{aligned}$$

When inequalities (6.1) hold, we succinctly denote this transfer by $L {\mathop {\longleftarrow }\limits ^{c}} H$.

### Proof of Property 4

Since $L< M < H$, the increase in *L*’s income affects the index

$$\begin{aligned} \Psi _{1,n}= 1- {1\over \lfloor n/2 \rfloor } \sum _{k=1}^{\lfloor n/2 \rfloor }{X_{k:n} \over X_{M:n}} \end{aligned}$$

because

$$\begin{aligned} {X_{L:n} \over X_{M:n} } < {X_{L:n}+c \over X_{M:n}}, \end{aligned}$$

whereas the decrease in *H*’s income does not affect $\Psi _{1,n}$ because *H* is not among the terms making up the definition of the index. Hence, $\Psi _{1,n}(\textbf{X})> \Psi _{1,n}(\textbf{X}')$. $\square$

### Proof of Property 5

When $M<L<H$, the index $\Psi _{1,n}$ is not affected by the transfer $L {\mathop {\longleftarrow }\limits ^{c}} H$ because, mathematically speaking, *L* and *H* are outside the summation range due to $\lfloor n/2 \rfloor \le M$ and, according to property (6.1), the transfer does not change the ordering of incomes. In other words, the median income and the incomes below it are not affected by the transfer, and we therefore have $\Psi _{1,n}(\textbf{X})= \Psi _{1,n}(\textbf{X}')$.

When $L<H< M$, both *L* and *H* are among the terms in the sum making up the definition of $\Psi _{1,n}$. Since we have the equations

$$\begin{aligned} {X_{L:n} \over X_{M :n}}+ {X_{H:n} \over X_{M :n}}&={X_{L:n}+c \over X_{M :n}}+ {X_{H:n}-c \over X_{M :n}}\\&= {X'_{L:n} \over X'_{M :n}}+ {X'_{H:n} \over X'_{M :n}} \end{aligned}$$

the value $\Psi _{1,n}$ is not affected by the transfer $L {\mathop {\longleftarrow }\limits ^{c}} H$. This implies $\Psi _{1,n}(\textbf{X})= \Psi _{1,n}(\textbf{X}')$ and establishes Property 5. $\square$

### Proof of Property 6

Consider first the case when $L< M < H$. Since $L< M$, we have $L\le \lfloor n/2 \rfloor$, and so the index

$$\begin{aligned} \Psi _{2,n}= 1- {1\over \lfloor n/2 \rfloor } \sum _{k=1}^{\lfloor n/2 \rfloor } {X_{k:n} \over X_{M+k:n}}. \end{aligned}$$

is affected by the transfer $L {\mathop {\longleftarrow }\limits ^{c}} H$ because

$$\begin{aligned} {X_{L:n} \over X_{M +L :n}}+{X_{H-M :n} \over X_{H:n}}&< {X_{L:n}+c \over X_{M +L :n}}+{X_{H-M :n} \over X_{H:n}-c },\\&= {X'_{L:n} \over X'_{M +L :n}}+{X'_{H-M :n} \over X'_{H:n}} \end{aligned}$$

which implies $$\Psi _{2,n}(\textbf{X})> \Psi _{2,n}(\textbf{X}')$$.

When $L<H< M$, we have $L<H \le \lfloor n/2 \rfloor$, and so

$$\begin{aligned} {X_{L:n} \over X_{M +L :n}} + {X_{H:n} \over X_{M +H :n}}&<{X_{L:n}+c \over X_{M +L :n}} + {X_{H:n}-c \over X_{M +H :n}}\\&= {X'_{L:n} \over X'_{M +L :n}} + {X'_{H:n} \over X'_{M +H :n}}, \end{aligned}$$

with the inequality holding because $X_{M +L:n}<X_{M +H:n}$. Hence, $\Psi _{2,n}(\textbf{X})> \Psi _{2,n}(\textbf{X}')$, thus concluding the proof of Property 6.

### Proof of Property 7

Since $M<L<H$, the incomes of *L* and *H* are above the median $X_{M:n}$, and so there are two *k*’s in the sum in the definition of $\Psi _{2,n}$ that give $M+k=L$ and $M+k=H$, respectively. Consequently, $\Psi _{2,n}(\textbf{X})> \Psi _{2,n}(\textbf{X}')$ holds if and only if the following inequality holds:

$$\begin{aligned} {X_{L-M :n} \over X_{L:n}}+{X_{H-M :n} \over X_{H:n}}&< {X_{L-M :n} \over X_{L:n}+c }+{X_{H-M :n} \over X_{H:n}-c },\\&= {X'_{L-M :n} \over X'_{L:n}}+{X'_{H-M :n} \over X'_{H:n}} . \end{aligned}$$

Simple algebra shows that the inequality is equivalent to $c> c_2$, where $c_2$ is defined by Eq. (5.2). This establishes Property 7. $\square$

### Proof of Property 7

(Continuation of Example 2) Alternatively, without delving into the psychology of people participating in various transfers and thus the plausibility of such transfers, we can have the following steps, some of which involving two well-off persons and some involving both well-off and struggling persons, leading to the same end-result (4, 5, 6, 7, 9, 18, 21) as in the first part of Example 2:

1. $W_1 {\mathop {\longleftarrow }\limits ^{3}} W_2$ results in (1, 3, 5, 7, 13, 17, 24) with $\Psi _{2,n}=0.8461$
2. $W_2 {\mathop {\longleftarrow }\limits ^{3}} W_3$ results in (1, 3, 5, 7, 13, 20, 21) with $\Psi _{2,n}=0.8450$
3. $S_3{\mathop {\longleftarrow }\limits ^{1}} W_2$ results in (1, 3, 6, 7, 13, 19, 21) with $\Psi _{2,n}=0.8265$
4. $S_2{\mathop {\longleftarrow }\limits ^{1}} W_2$ results in (1, 4, 6, 7, 13, 18, 21) with $\Psi _{2,n}=0.8050$
5. $S_2 {\mathop {\longleftarrow }\limits ^{1}} W_1$ results in (1, 5, 6, 7, 12, 18, 21) with $\Psi _{2,n}=0.7844$
6. $S_1{\mathop {\longleftarrow }\limits ^{3}} W_1$ results in (4, 5, 6, 7, 9, 18, 21) with $\Psi _{2,n}=0.6640$

Step 1 is justified by our earlier argument at the beginning of this example saying that any transfer higher than 2 but less than 5 from $W_2$ to $W_1$ is legitimate, and we transfer $c=3$. To justify Step 2, we note that we can only transfer less than $(24-17)/2=3.5$ but more than $(3\times 24^2 - 5\times 17^2) /(3\times 24+ 5\times 17)= 1.8025$, and so we transfer $c=3$. All Steps 3–6 are from well-off persons to struggling ones, and so the only requirement on the transfers is that they should maintain the original ordering of incomes.

### Proof of Property 8

The transfer $L {\mathop {\longleftarrow }\limits ^{c}} H$ affects the index

$$\begin{aligned} \Psi _{3,n}= 1- {1\over \lfloor n/2 \rfloor } \sum _{k=1}^{\lfloor n/2 \rfloor } {X_{k:n} \over X_{n-k+1:n}} \end{aligned}$$

via both *L* and *H* because

$$\begin{aligned} {X_{L:n} \over X_{n-L+1:n}}+ {X_{n-H+1:n} \over X_{H:n}}&< {X_{L:n}+c \over X_{n-L+1:n}}+ {X_{n-H+1:n} \over X_{H:n}-c } \\&= {X'_{L:n} \over X'_{n-L+1:n}}+ {X'_{n-H+1:n} \over X'_{H:n}}, \end{aligned}$$

which implies $\Psi _{3,n}(\textbf{X})> \Psi _{3,n}(\textbf{X}')$ and establishes Property 8.

### Proof of Property 9

Consider first the case of two well-off persons, that is, $M<L<H$. Since $\lfloor n/2 \rfloor + \lceil n/2 \rceil =n$ and $M=\lceil n/2 \rceil$, we have $n-k+1>M$ for every $k\le \lfloor n/2 \rfloor$. Consequently, there are two *k*’s in the sum in the definition of $\Psi _{3,n}$ that give $n-k+1=L$ and $n-k+1=H$, respectively, because $M<L<H$. Hence, the inequality $\Psi _{3,n}(\textbf{X})\ge \Psi _{3,n}(\textbf{X}')$ holds if and only if

$$\begin{aligned} {X_{n-L+1:n} \over X_{L:n}}+{X_{n-H+1:n} \over X_{H:n}}&\le {X_{n-L+1:n} \over X_{L:n}+c }+{X_{n-H+1:n} \over X_{H:n}-c },\\&= {X'_{n-L+1:n} \over X'_{L:n}}+{X'_{n-H+1:n} \over X'_{H:n}} \end{aligned}$$

which is equivalent $c\ge c_3$, where

$$\begin{aligned} c_3={ X_{n-L+1:n}X_{H:n}^2-X_{n-H+1:n} X_{L:n}^2\over X_{n-L+1:n}X_{H:n}+X_{n-H+1:n}X_{L:n}}. \end{aligned}$$

Recall now that the transfer $L {\mathop {\longleftarrow }\limits ^{c}} H$ does not change the ordering of incomes, and thus we must have $X_{L:n}+c < X_{H:n}-c$, which is equivalent to $c<c_0$, where

$$\begin{aligned} c_0={ X_{H:n}-X_{L:n} \over 2}. \end{aligned}$$

Hence, to have $L {\mathop {\longleftarrow }\limits ^{c}} H$ for some $c>0$, we must have $c_3<c_0$, which is equivalent to

$$\begin{aligned} 2\big ( X_{n-L+1:n}X_{H:n}^2-X_{n-H+1:n}X_{L:n}^2\big )< \big (X_{H:n}-X_{L:n}\big )\big ( X_{n-L+1:n}X_{H:n}+X_{n-H+1:n}X_{L:n}\big ), \end{aligned}$$

which simplifies to

$$\begin{aligned} X_{n-L+1:n}X_{H:n}^2-X_{n-H+1:n}X_{L:n}^2 < \big ( X_{n-H+1:n} - X_{n-L+1:n}\big ) X_{L:n}X_{H:n}. \end{aligned}$$

The latter inequality is impossible because $L<H$ implies $X_{H:n}>X_{L:n}$ and $X_{n-H+1:n} < X_{n-L+1:n}$. Consequently, it is impossible to have $c_3<c_0$ and so there is not a single $c>0$ that satisfies $c\ge c_3$ and $c<c_0$ simultaneously. This shows that the only possibility that exists is $\Psi _{3,n}(\textbf{X})< \Psi _{3,n}(\textbf{X}')$.

Consider now the case of two struggling persons, that is, $L<H< M$. In this case we have $L<H\le \lfloor n/2 \rfloor$ and so $L {\mathop {\longleftarrow }\limits ^{c}} H$ affects $\Psi _{3,n}$ because of the inequality

$$\begin{aligned} {X_{L:n} \over X_{n-L+1:n}} + {X_{H:n} \over X_{n-H+1:n}}&> {X_{L:n}+c \over X_{n-L+1:n}} + {X_{H:n}-c \over X_{n-H+1 :n}}\\&= {X'_{L:n} \over X'_{n-L+1:n}} + {X'_{H:n} \over X'_{n-H+1:n}} \end{aligned}$$

that holds due to $X_{n-L+1:n} > X_{n-H+1:n}$. Hence, $\Psi _{3,n}(\textbf{X})< \Psi _{3,n}(\textbf{X}')$, concluding the proof of Property 9.
